# Human IgE monoclonal antibody recognition of mite allergen Der p 2 defines structural basis of an epitope for IgE cross-linking and anaphylaxis *in vivo*

**DOI:** 10.1093/pnasnexus/pgac054

**Published:** 2022-06-02

**Authors:** Kriti Khatri, Crystal M Richardson, Jill Glesner, Anyway Brenda Kapingidza, Geoffrey A Mueller, Jian Zhang, Cole Dolamore, Lisa D Vailes, Sabina Wünschmann, R Stokes Peebles, Martin D Chapman, Scott A Smith, Maksymilian Chruszcz, Anna Pomés

**Affiliations:** Department of Chemistry and Biochemistry, University of South Carolina, Columbia, SC 29208, USA; InBio, Charlottesville, VA 22903, USA; InBio, Charlottesville, VA 22903, USA; Department of Chemistry and Biochemistry, University of South Carolina, Columbia, SC 29208, USA; Duke Human Vaccine Institute, Duke University School of Medicine, Durham, NC 27710, USA; Genome Integrity and Structural Biology Laboratory, National Institute of Environmental Health Sciences, Research Triangle Park, NC 27709, USA; Department of Medicine, Vanderbilt University Medical Center, Nashville, TN 37232, USA; Department of Chemistry and Biochemistry, University of South Carolina, Columbia, SC 29208, USA; InBio, Charlottesville, VA 22903, USA; InBio, Charlottesville, VA 22903, USA; Department of Medicine, Vanderbilt University Medical Center, Nashville, TN 37232, USA; InBio, Charlottesville, VA 22903, USA; Department of Medicine, Vanderbilt University Medical Center, Nashville, TN 37232, USA; Department of Chemistry and Biochemistry, University of South Carolina, Columbia, SC 29208, USA; InBio, Charlottesville, VA 22903, USA

**Keywords:** IgE antibody, allergy, allergen–antibody interaction, house dust mite, anaphylaxis

## Abstract

Immunoglobulin E (IgE) antibody is a critical effector molecule for adaptive allergen-induced immune responses, which affect up to 40% of the population worldwide. Allergens are usually innocuous molecules but induce IgE antibody production in allergic subjects. Allergen cross-linking of IgE bound to its high affinity receptor (FcεRI) on mast cells and basophils triggers release of histamine and other mediators that cause allergic symptoms. Little is known about the direct allergen–IgE antibody interaction due to the polyclonal nature of serum IgE and the low frequency of IgE-producing B cells in blood. Here, we report the X-ray crystal structure of a house dust mite allergen, Der p 2, in complex with Fab of a human IgE monoclonal antibody (mAb) isolated by hybridoma technology using human B cells from an allergic subject. This IgE mAb, 2F10, has the correct pairing of heavy and light chains as it occurs *in vivo*. Key amino acids forming the IgE epitope on Der p 2 were identified. Mutation of these residues ablated their functional ability to cross-link IgE in a mouse model of passive systemic anaphylaxis. These analyses revealed an important conformational epitope associated with the IgE antibody repertoire to a major mite allergen.

Significance Statement Immunotherapy is an allergen-specific disease-modifying treatment that involves administration of increasing allergen doses to reduce IgE production, but can induce side-effects due to IgE cross-linking by allergen. Direct knowledge of IgE epitopes that bind to specific allergens will contribute to designing engineered allergens with impaired IgE reactivity resulting on diminished side-effects. The direct identification of critical, allergen-binding IgE epitopes, such as the one reported here, thanks to IgE hybridoma technology, has not been possible before due to the low frequency of IgE-producing B cells in blood. A direct mapping of IgE antibodies by X-ray crystallography will provide high resolution information of allergen epitopes bound by IgEs from the human repertoire and facilitate structure-based design of allergen vaccines.

**Abbreviations**
CD: Circular dichroism
CDR: Complementarity determining region
D_2_O: Deuterated water
Fab: Fragment antibody binding
FcɛRI: High-affinity IgE receptor
IMAC: Immobilized metal affinity chromatography
IgE: Immunoglobulin E
mAb: Monoclonal antibody
PBS: Phosphate Buffered Saline
DSS: 4,4-dimethyl-4-silapentane-1-sulfonic acid
rDer p 2: Recombinant Der p 2
nDer p 2: Natural Der p 2
NMR: Nuclear Magnetic Resonance
PDB: Protein Data Bank
rmsd: Root-mean-square deviation
Three letter code (e.g. Asp): Single amino acid
SD: Standard deviation
SHM: Somatic hypermutations
Single letter code and number (e.g. K100D): Mutation

## Introduction

Allergy is an increasingly important disease worldwide triggered by the development in susceptible individuals of an adverse Immunoglobulin E (IgE) immune response to molecules (allergens) that are otherwise innocuous in a nonallergic population (1). The interaction between IgE antibodies and the corresponding areas they recognize on the allergen, or epitopes, are key for the development of the allergic response. In the process of sensitization, allergen-specific T helper 2 cells are primed to produce cytokines (IL-4 and IL-13) that induce antibody class switching to epsilon immunoglobulin heavy chain, followed by IgE production by B cells (2). Subsequently, IgE antibodies bind to high-affinity receptors (FcɛRI) on the surface of mast cells and basophils. Allergen interaction with these mast cell- and basophil-bound IgEs will induce cell degranulation upon cross-linking of the IgE–FcɛRI complexes, leading to the release of mediators (such as histamine, lipid mediators, chemokines, and cytokines) that will cause the immediate phase of the allergic reaction (2). The allergen–IgE interaction also contributes to allergen uptake by antigen presenting cells (dendritic cells and monocytes) and presentation of allergen-derived peptides to specific CD4+ T cells, which drive the late phase allergic response (2). However, it has been more than 50 years since the discovery of IgE in 1966-67 (3, 4), and there have been no reported structures of IgE (with the pairing of heavy and light chains that occurs *in vivo*) in complex with an allergen (3, 4).

Various methods have been used historically to map IgE-binding allergen epitopes indirectly. Early studies identified linear or continuous epitopes, by testing IgE antibody binding to linear peptides or recombinant allergen fragments and performing mutagenesis analyses (5, 6). Linear epitopes are composed of contiguous amino acids in the protein. They are common in foods because allergens are processed and proteolytically cleaved in the digestive tract before the peptides are exposed to the immune system. However, most IgE are expected to recognize conformational epitopes, especially on aeroallergens (7). These are discontinuous epitopes, formed by residues from different parts of the sequence that are brought together by the folding of the protein (8). Therefore, the interactions between the part of the antibody that recognizes the allergen, or paratope, and the epitope on the allergen are only fully deciphered if the structure of the IgE–allergen complex is known. To date, structures of IgE–allergen complexes (with IgE derived from mite-allergic subjects and with correct pair of heavy and light chains) have not been reported yet. The main challenge to directly identifying IgE epitopes has been the polyclonal nature of IgE and its low concentration in blood (ng/mL). These aspects hinder crystallographic studies, owing to the requirement for high amounts of pure and homogeneous protein. Sequencing of IgE antibodies has also been problematic, due to the low numbers of antigen-specific B cells that can be isolated from the blood (3 × 10^−7^ to 7 × 10^−6^) (9, 10). An alternative to using IgE in crystallographic studies, is to use murine IgG monoclonal antibodies (mAbs) as surrogates of human IgE to identify IgE antibody binding sites. This involves determining X-ray crystal structures of allergens in complex with murine IgG mAb constructs that inhibit human IgE antibody binding to the allergen, followed by detailed site-directed mutagenesis to identify amino acids involved in the IgE epitope (11–18). By a similar approach, the closest picture to an IgE epitope was obtained from X-ray crystal structures of the cow's milk allergen, Bos d 5, and timothy grass pollen, Phl p 2, in complex with specific antibody constructs isolated from IgE phage display combinatorial libraries (19, 20). These studies involved combining IgE antibody heavy chains with light chains from the same or a different subject, to form IgE antibody constructs, which were displayed by phagemids and selected by their allergen specificity. However, such heavy–light chain combinations are not necessarily the same as ones that occur *in vivo*(21). In contrast, a crystal structure of a human (IgG) antibody/allergen complex with an antibody derived from a single B cell of an allergic individual, and thus with naturally matched heavy and light chains, has been reported (22).

Der p 2 is one of the most important major allergens from the house dust mite species *Dermatophagoides pteronyssinus*. This allergen induces IgE sensitization in ≥ 79% of mite-allergic subjects in temperate areas of the world, such as the United States, resulting in the development of allergic diseases such as atopic dermatitis and asthma (1, 23, 24). Despite this, the structural basis of its interaction with human IgE remains unresolved. Antibody epitopes of Der p 2 for several murine IgG mAbs were originally identified in 2001 by hydrogen-exchange protection nuclear magnetic resonance (NMR) (25). The epitope for one of these IgG mAbs (7A1) was subsequently identified by determining the X-ray crystal structure of recombinant Der p 2 (rDer p 2) in complex with the fragment antibody-binding (Fab) region of 7A1 (26). Mutagenesis analysis of this 7A1 epitope led to the identification of binding sites recognized by IgE antibody constructs that were obtained from a phage display library constructed from peripheral blood mononuclear cells of a mite-allergic patient (26). Recently, the binding sites of four anti-Der p 2 human IgE mAbs isolated by hybridoma technology, including the IgE mAb 2F10, were identified by NMR and immunoassays (27). While NMR defines only few labeled amino acids involved in the IgE epitope, X-ray crystallography provides a more detailed picture of the allergen–antibody interaction (18, 28).

Here, we describe a major milestone by reporting the X-ray crystal structure of an allergen, Der p 2, in complex with an IgE antibody construct derived from a human IgE-producing B cell. This was accomplished by using a human IgE mAb comprising the correct pairing of heavy and light chains obtained by human hybridoma technology (27, 29). B cells expressing allergen-specific IgE that were isolated from blood were fused with a myeloma partner to confer immortality to the resulting hybridoma. Antibody constructs with IgE variable domains were then recombinantly expressed to determine their structure in complex with the allergen. Our multifaceted strategy involved detailed site-directed mutagenesis analyses, coupled with *in silico* and *in vivo*assessments of IgE antibody binding using a mouse model of passive anaphylaxis, in order to verify the IgE epitope and its relevance to the allergic response. The collective findings represent a significant advance in the field of allergen–antibody research that will provide structural basis for rational design of allergen vaccines for new treatments of allergic disease.

## Results

### The structure of Der p 2 in complex with IgE mAb 2F10 Fab reveals that most of the antibody–allergen interface involves the heavy chain

**Fig. 1.**
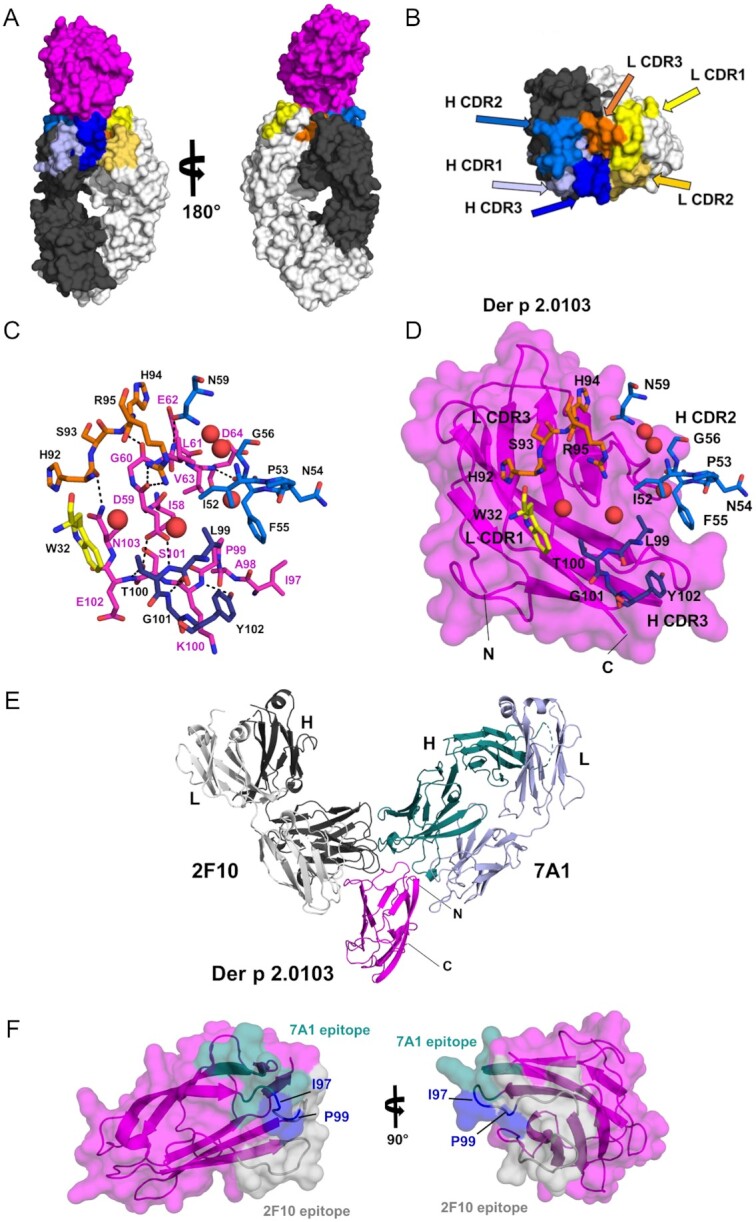
X-ray crystal structure of Der p 2 in complex with Fab of the IgE mAb 2F10. (A) Overall structure of Der p 2.0103 in complex with IgE mAb 2F10 Fab, shown in space filling representation. Der p 2 is shown in magenta, whereas light and heavy chains of the Ab are marked in light and dark gray, respectively. (B) The Complementarity Determining Regions (CDRs) of the heavy and light chains of the antibody are mapped on the molecular surface of IgE mAb 2F10 Fab, colored as in section A and labeled. (C) Residues forming the epitope and the paratope, responsible for allergen–antibody interactions are presented in stick representation and labeled in magenta and black, respectively. Water molecules buried between the allergen and the antibody are shown as red spheres. Colors are consistent with the images in (A) and (B). (D) Same area and molecular orientation as in (C), with the addition of Der p 2.0103 (magenta), shown in space filling and cartoon representations. Several 2F10 residues, including the ones responsible for interactions with the allergen, are presented in stick representation and labeled in black. (E) Superposition of the human 2F10 IgE mAb and the murine 7A1 IgG mAb Fabs binding to Der p 2.0103 to assess the relative location of their epitopes, shown in (F). (F) Surface of Der p 2.0103 with 7A1 and 2F10 epitopes marked in teal and gray, respectively. Ile97 and P99 (marked in blue) belong to both epitopes. The model of the allergen is derived from the Der p 2.0103–2F10 Fab complex structure.

The structure of a common Der p 2 variant (Der p 2.0103) in complex with the Fab of IgE mAb 2F10 was determined at 2.1 Å resolution (Fig. 1A and B; Supplementary Material Appendix, Table S1, Supplemental Results for additional details). The epitope–paratope interface between Der p 2.0103 and 2F10 Fab has an area of 749 Å^2^, according to the software PDBePISA (30). Approximately, 72% of the interface area corresponds to the region between the allergen and the heavy chain of the antibody, revealing the importance of the heavy chain in the allergen–antibody interaction (30). The antibody recognizes an epitope that includes residues 58 to 64 and 97 to 103 of the allergen (Fig. 1C, D). The IgE mAb 2F10 was found to be moderately mutated versus the germline (Supplementary Material Appendix, Table S2). The sequence of the IgE mAb 2F10 differs from the germline sequence in: (1) eight and six somatic hypermutations (SHM) in the heavy and light chains, respectively, (2) three additional mutations in the junction (CDR3) of each of the chains (which could be due to a recombination event instead of SHM), and (3) two amino acid deletions in the heavy chain. From these 20 mutations, only four residues are involved in forming hydrogen bonds with Der p 2 (Gly101 and Tyr102 from the heavy chain, and His92 and His94 from the light chain), therefore, directly contributing to binding the allergen (Supplementary Material Appendix, Supplemental Results for additional details).

The binding affinity of the IgE mAb 2F10 for Der p 2 was measured by surface plasmon resonance. This affinity, reported as the equilibrium dissociation constant (K_D_), was 4.74E-10 M as measured by full kinetics and 2.97E-10 M as measured by scouting analysis. These K_D_ show a high affinity of the allergen–antibody interaction consistent with what is expected of a somatically hypermutated (and class-switched) antibody.

Interestingly, PDBePISA also suggests that an assembly in which two Fab fragments binding to a putative Der p 2.0103 dimer may be stable in solution. Analysis of such assembly indicates that the interface area between Der p 2.0103 molecules has approximately 745 Å^2^. Der p 2 is present in solution as both monomers (mostly) and dimers at high allergen concentration (Supplementary Material Appendix, Figure S1A). Moreover, the putative dimeric form that was observed in the crystal of 2F10–Der p 2.0103 complex is almost identical to the one observed in the crystal of Der p 2 alone, with an area of ∼673 Å^2^ (PDB code: 1KTJ). The dimers superpose with a rmsd value of 0.7 Å over 258 superposed residues (Supplementary Material Appendix, Figure S1B).

The interactions between the antibody and antigen are mediated by 11 hydrogen bonds (Supplementary Material Appendix, Table S3) and multiple hydrophobic interactions. Der p 2.0103 Asp59 is heavily involved in formation of H-bonds (two with the heavy chain and two with the light chain of the antibody). Interestingly, only side chains of Asp59 and Asn103 on Der p 2.0103 are involved in the H-bonds network, and the remaining epitope atoms participating in formation of the H-bonds originate from the main chain of the allergen. In addition, several water molecules are trapped between the allergen and antibody (Fig. 1C, D), and improve the fit of the molecular interfaces, as well as bridge the protein molecules though hydrogen bonds.

Major hydrophobic interactions between the allergen and the antibody are mediated by residues Ile58, Leu61 (that interacts with both heavy and light chains), Val63, Ile97, Pro99, and Lys100 from the epitope. These interactions, which involve the largest area, correspond to a region of the epitope that is in contact with the heavy chain of the 2F10 antibody.

On the paratope side, residues participating in formation of H-bonds originate mainly from Complementarity Determining Regions (CDRs) 2 and 3 of the heavy chain, and CDR3 of the light chain, whereas the remaining CDRs do not form any major contacts with the allergen. The largest areas of hydrophobic contacts with the allergen are mediated by Ile52, Phe55, and Leu99 from the heavy chain of the antibody.

### Comparison of IgE–Der p 2 complex with other non-IgE–allergen complexes

Next, we sought to analyze how the structure of the IgE–Der p 2 interaction compared with non-IgE antibodies in complex with allergens, to gain further insight into the biological basis of IgE. Currently, only one crystal structure of Der p 2 in complex with a non-IgE mAb has been determined, which is that containing the Fab fragment of murine IgG 7A1 (Protein Data Bank—PDB—code: 6OY4) (26). Superposition of our current IgE 2F10 model with the IgG 7A1 model (Fig. 1E and F) indicates that 2F10 and 7A1 epitopes are adjacent, with very small overlap, which did not prevent simultaneous binding of both antibodies by two-site ELISA (27). Most likely in this case, a slight adjustment in the conformation of a small number of antibody residues is enough to avoid steric clashes. This overlap included residues Ile97 and Pro99 of Der p 2.0103 that participate in hydrophobic allergen–antibody interactions (Supplementary Material Appendix, Supplemental Results for additional details). Der p 2 is a flexible molecule and can adopt different conformations upon antibody binding, as previously reported (26).

**Fig. 2.**
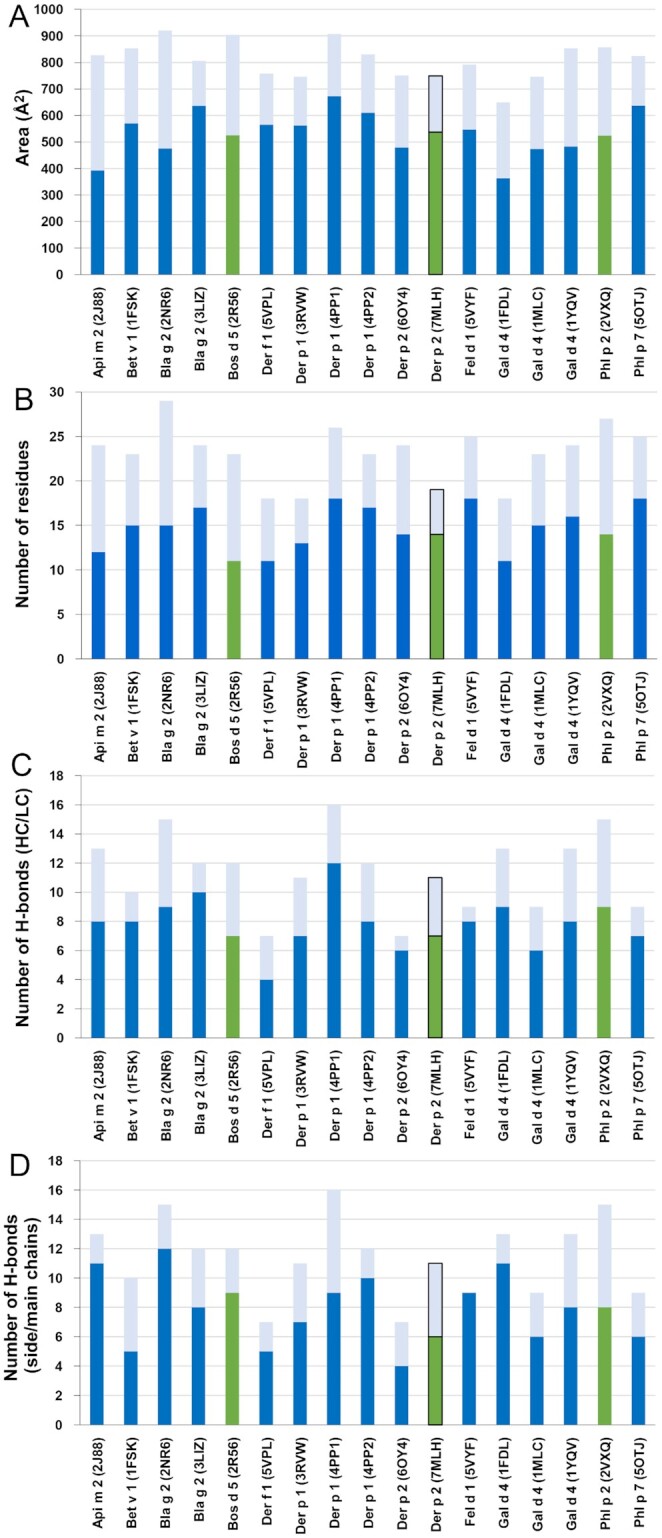
Analysis of allergen–antibody interactions in 17 currently determined complexes comprising 13 murine IgG mAb constructs, a human IgG derived from a single B cell of an allergic individual, two Fab derived from IgE combinatorial libraries (specific for Bos d 5 or Phl p 2), and the human IgE mAb 2F10. For all the plots, complexes of allergens with IgG or IgE variable domains are marked in blue or green, respectively. The border of the bar corresponding the Der p 2–2F10 Fab complex was outlined in the four plots. For Phl p 7 (PDB code: 5OTJ), the area corresponding to the conventional mode of binding (not the superantigen-like recognition also reported for this structure) is presented (22). (A) Allergen–antibody interface areas. Dark colors mark the area of the interface that corresponds to the heavy chain and light color indicates the area of interaction with the light chain (22). (B) Number of residues from heavy chain (dark colors) and light chain (pale color) that were identified by PDBePISA as forming contacts with allergens (30). (C) Number of hydrogen bonds between paratopes and epitopes in the antibody–allergen complexes. Hydrogen bonds formed by heavy chain residues are indicated with dark colors, while hydrogen bonds formed by light chain residues are marked with pale colors. (D) H-bonds with side versus main chains. Dark colors indicate hydrogen bonds involving side chains of antibodies, while pale colors mark hydrogen bonds formed by main chain atoms of the antibodies.

To assess how IgE binds to the allergen in comparison with other known antibody constructs, the structural characteristics of the epitope and paratope observed in the 2F10–Der p 2.0103 complex were compared with those of 16 other antibodies (13 murine IgG mAbs, a human IgG derived from a single B cell of an allergic individual, and two IgE constructs from combinatorial libraries made from allergic patients) in complex with their specific allergen that have their structures determined (18). According to this comparison, the overall interface area between 2F10 and Der p 2.0103 is slightly smaller than the average interface area (810 Å^2^) observed for the 17 antibody–allergen complexes (Fig. 2A). Similar to other interfaces, the heavy chain of the antibody is responsible for most of the contacts. However, the number of residues in the paratope is relatively small (Fig. 2B), which is similar to the paratope of the cross-reactive IgG mAb 4C1 recognizing Der f 1 or Der p 1 (PDB codes 5VPL and 3RVW, respectively), as well as the paratope of the D1.3 antibody binding to lysozyme (PDB code: 1FDL). Most structural elements were within the same range for IgE–Der p 2 and the other 16 allergen–antibody complexes (Supplementary Material Appendix, Supplemental Results for additional details). The structural elements include number of paratope residues in the heavy and light chains (Fig. 2B), hydrogen bonds with each of both chains (Fig. 2C), hydrogen bonds with the antibody side and main chains (Fig. 2D), and the secondary elements in the epitope (which were typically strands and turns; Supplementary Material Appendix, Figure S2). The patterns of hydrogen bond formation according to the involvement of side and main chains of the allergen and the antibody were further analyzed and were variable, with no unique preference for the 2F10 IgE mAb (Supplementary Material Appendix, Figure S3).

### IgE mAb 2F10 also recognizes homologous allergens from other house dust mite species, but not storage mites

To assess if the 2F10 IgE mAb can cross-react with homologous allergens from phylogenetically related mite species, sequence homologies were analyzed at the epitope level (Supplementary Material Appendix, Figure S4A and B) and IgE antibody binding was tested to several group 2 mite allergens. These included house dust mite allergens from the same Pyroglyphidae family (*D. pteronyssinus*, *D. farinae*, and *Euroglyphus maynei*), as well as homologous allergens from storage mites of the Glycyphagoidea superfamily (*Glycyphagus domesticus* and *Lepidoglyphus destructor*) that share lower amino acid sequence identities with Der p 2 (< 40%; Supplementary Material Appendix, Figure S4C, Supplemental Results for additional details). The IgE mAb 2F10 bound to recombinant dust mite allergens Der p 2.0101, Der p 2.0103, Der f 2.0103, and Eur m 2.0101, but not to storage mite allergens Gly d 2.0101 and Lep d 2.0101, in agreement with lower amino acid sequence identities at the epitope level (Supplementary Material Appendix, Figure S5).

### Rational design of site-directed mutagenesis to modify the 2F10 epitope on Der p 2

Structural analysis revealed that two residues in the epitope interact with both the heavy and light chains of IgE mAb 2F10: Asp59 establishes hydrogen bonds with each chain (Aps59 main chain with the light chain), and Leu61 establishes hydrophobic interactions. Due to its dual interaction with both chains, Asp59 and Leu61 were selected for site-directed mutagenesis, with the goal to significantly reduce IgE mAb 2F10 binding. Additional residues that establish main chain hydrogen bonds with the antibody are: Glu62, Asp64, Lys100, and Glu102 with the heavy chain, and Gly60 with the light chain (Supplementary Material Appendix, Table S3). However, these were not selected for site-directed mutagenesis, which alters the side chain of a residue, and in most cases, it does not impact interactions (like hydrogen bonds) involving only main chain atoms of residues in the epitope.

Therefore, Asp59 and Leu61 were changed originally to alanine, replacing longer side chains with methyl groups, with the goal to reduce the number of H-bonds in the epitope–paratope interface for Asp59, and to reduce hydrophobic interactions for Leu61. These two substitutions were applied either: (1) to the wildtype allergen Der p 2.0103, resulting in *mutant #1* (rDer p 2 D59A L61A), or (2) to Der p 2.0103 containing mutations in the adjacent IgG mAb 7A1 epitope (7A1 mut) resulting in *mutant #2* (D59A L61A + 7A1 mut). The latter contains two substitutions (K96E-I97E) in the 7A1 epitope that is adjacent to the epitope for IgE mAb 2F10, which were geared to perturb a hydrogen bond network and change to the hydrophilic and negatively charged residue Glu. These substitutions were previously shown to prevent binding of IgG mAb 7A1 and binding of an IgE antibody construct obtained from a phage display library (26).

**Fig. 3.**
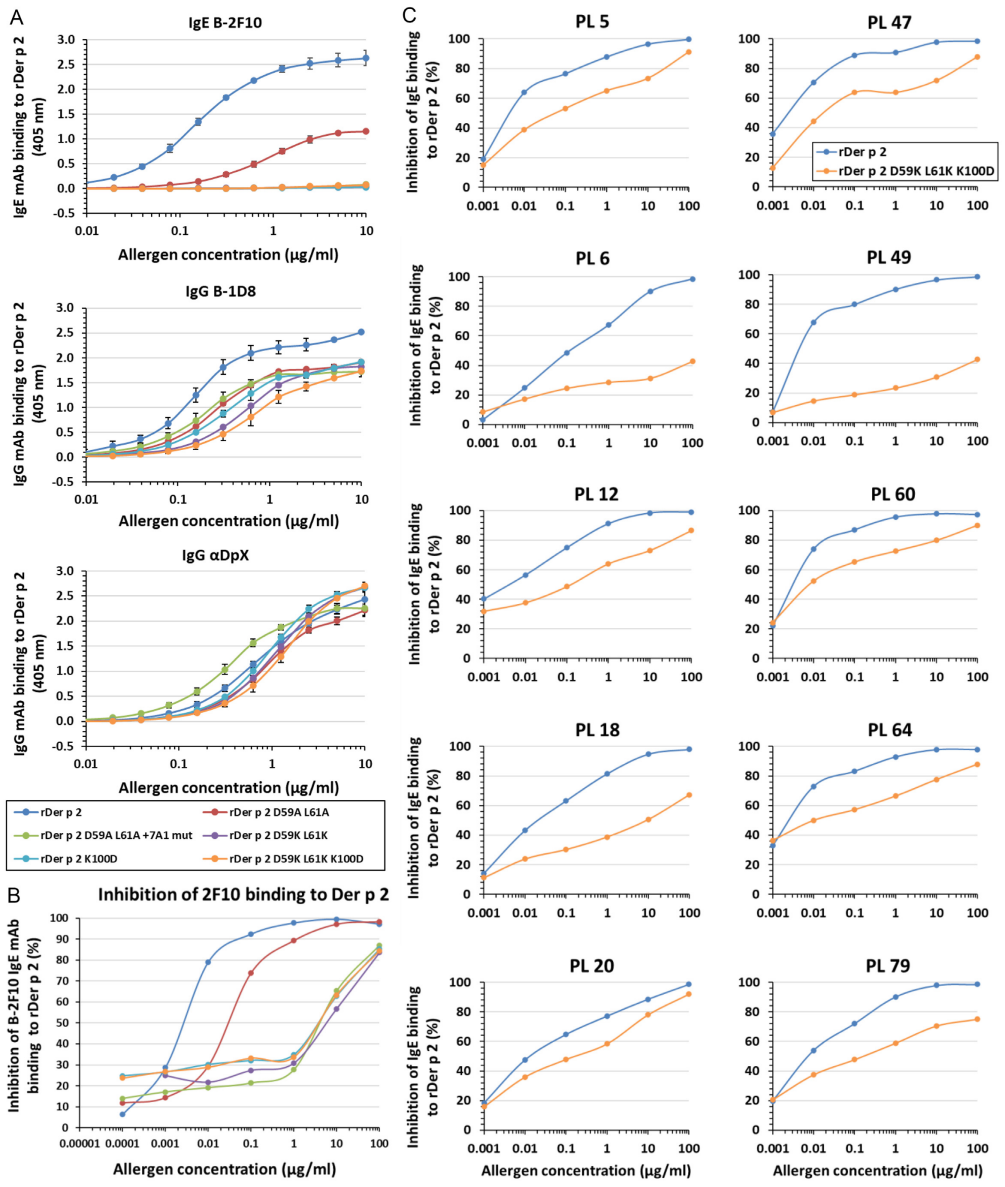
Mutagenesis analysis of the 2F10 IgE mAb epitope. (A) Dose–response curves of human IgE or mouse IgG mAb binding to rDer p 2. Mutations to the 2F10 epitope on Der p 2 decreased or eliminated binding of the biotinylated (B)-2F10 human IgE mAb. The capacity of the mutants to bind murine IgGs B-ID8 and αDpX was retained, indicating that they were correctly folded. Data is representative of three experiments, with average of 2 ± SD. Figure legend also applies to (B) and (C). (B) Inhibition of human IgE mAb 2F10 binding to Der p 2 by epitope mutants. There was up to 2,200-fold reduction of the inhibition of B-2F10 binding to rDer p 2 by the epitope mutants compared to the wildtype. Plot is representative of two to three experiments, in duplicate ± SD. (C) Comparison of the wildtype and the 2F10 epitope mutant using plasma from mite-allergic subjects. Inhibition of polyclonal IgE antibody binding to Der p 2 by Der p 2 2F10 epitope triple mutant was reduced versus the wildtype for 10 mite-allergic subjects sensitized to Der p 2. Plasma from the allergic subjects are designated by “PL.” Data are averages of duplicates ± SD.

A third mutant was designed to change Asp59 and Leu61 to lysine instead of alanine. These changes were targeted to impair the allergen–antibody interaction by replacing small side chains with larger ones, as well as changing a negatively charged aspartate and a neutral leucine to positively charged lysine (*mutant #3*). Another mutant was aimed at eliminating hydrophobic interactions by mutating the positively charged Lys100 to a negatively charged Asp that has a shorter side chain (*mutant #4*). Lys100 main chain residues establish main chain hydrogen bonds with the antibody, and therefore, the K100D mutation might not affect them. Finally, a 2F10 epitope triple mutant (*mutant #5*) was made by combining the substitutions in *mutants #3* and *4*. The K100D substitution was added to the D59K-L61K mutant to ensure that these three substitutions (that turned out to be effective at impairing IgE mAb 2F10 binding) were present for disruption of antibody binding. A summary of the substitutions in the mutants is shown in Supplementary Material Appendix, Table S4. All purified mutants (Supplementary Material Appendix, Figure S6) looked comparable to the wildtype on SDS-PAGE gel and were proven to have an overall fold as Der p 2.0103 wildtype by three approaches: (1) spectra of amide and methyl resonances by NMR (Supplementary Material Appendix, Figure S7), (2) circular dichroism (CD) spectra (Supplementary Material Appendix, Figure S8), and (3) their capacity to bind other antibodies (IgE mAbs 1D8 and αDpX) that recognize distant epitopes from the mutated one (Fig. 3A).

### Few amino acids in the epitope were essential for IgE mAb binding

The effect of the epitope mutations on 2F10 IgE mAb binding was analyzed. A reduction in maximum IgE mAb 2F10 binding to the mutant rDer p 2 D59A L61A and smaller slope of the curve versus the wildtype was observed by two-side immunoassays. The largest reductions were observed for the other four Der p 2 mutants, which displayed a flat dose–response curve (Fig. 3A). In agreement with these results, inhibition assays showed that the change of Asp59 and Leu61 to alanine led to an approximate 12-fold reduction (versus the wildtype) in the inhibition of 2F10 binding to rDer p 2, whereas significantly higher and similar reductions of up to 2,200-fold were observed for the other four mutants (Fig. 3B). As a control, the capacity of the epitope triple mutant to inhibit binding of 5D10 and 1B8 IgE mAb to Der p 2 was compared to the wildtype. Since these two IgE mAb are known to bind to a different epitope than that of 2F10, the triple mutant behaved in a similar way as the wildtype allergen, as expected from a correctly folded mutant (Supplementary Material Appendix, Figure S9).

### IgE mAb 2F10 contributes to the human polyclonal IgE response to Der p 2

First, we sought to establish the degree of biological relevance of the human IgE mAb 2F10, based on its contribution to human polyclonal IgE responses to Der p 2. This was accomplished by testing the ability of IgE mAb 2F10 (as well as two other IgE mAb, 2G1, and 1B8 as controls, all three expressed as hybrid IgE–IgG constructs) to inhibit binding of polyclonal IgE to Der p 2 in plasma obtained from allergic subjects who were highly sensitized to this allergen. The 2F10 antibody showed the largest inhibition among the three hybrid constructs tested, in 9 out of 10 specimens (21.4% mean inhibition; range 11.9% to 36.1%; *n* = 10), whereas 2G1 and 1B8 inhibited IgE binding by 10.1% (range 1.2 to 19.5) and 12.7% (range 3.7 to 21.8), respectively (Supplementary Material Appendix, Figure S10). These results indicate that IgE mAb 2F10 (or a similar IgE mAb with overlapping epitope specificity) is an important component of the human IgE polyclonal response to Der p 2, and that additional IgE clones, distinct from the one associated with 2F10, also contribute to this response (Supplementary Material Appendix, Figure S10, Supplemental Methods and Results for additional details).

Once the relevance of 2F10 IgE mAb regarding the human polyclonal IgE response to Der p 2 was established, a Der p 2 mutant with impaired capacity to bind 2F10 was assessed for its capacity to inhibit binding of polyclonal IgE from allergic patients to Der p 2. The *mutant #5* with the three combined amino acid substitutions in the 2F10 epitope (rDer p 2 D59K L61K K100D) was selected to compare with the wildtype allergen. For all 10 patients tested, there was reduced inhibition of IgE antibody binding to Der p 2 by the mutant versus the wildtype, highlighting the contribution of the 2F10 clone to the full polyclonal IgE antibody response (Fig. 3C). The effect was variable depending on the plasma (range 0.3 to 12.5-fold increase of IC50 for the mutant versus the wildtype; 4.5-fold in average). There was a significant correlation between the fold-change in IC50 of the mutant versus the wildtype and the % inhibition of the polyclonal IgE response by IgE mAb 2F10 (*r* = 0.83; *P* = 0.0027). For some plasma, the curve for the mutant did not reach a 100% inhibition as the one for the wildtype allergen (e.g. PL18, PL49), which indicates the immunodominance of the IgE recognizing the disrupted epitope versus the total IgE polyclonal response. In contrast, other plasma such as PL20 showed similar inhibition curves for wildtype and mutant, indicating the importance of other IgE mAb for that particular IgE polyclonal antibody response. In addition, a high and significant inverse correlation (*r* = −0.86; *P* = 0.0016) was found between the % inhibition of the polyclonal IgE binding to Der p 2 by the 2F10 epitope triple mutant and the IgE titers measured by ImmunoCAP (in KU_A_/L = IU/mL; Supplementary Material Appendix, Figure S11). The % inhibition was at the maximum concentration of inhibitor tested (100 µg/mL), as shown in Fig. 3(C).

### Reactivity of the epitope triple mutant in a mouse model of passive anaphylaxis

**Fig. 4.**
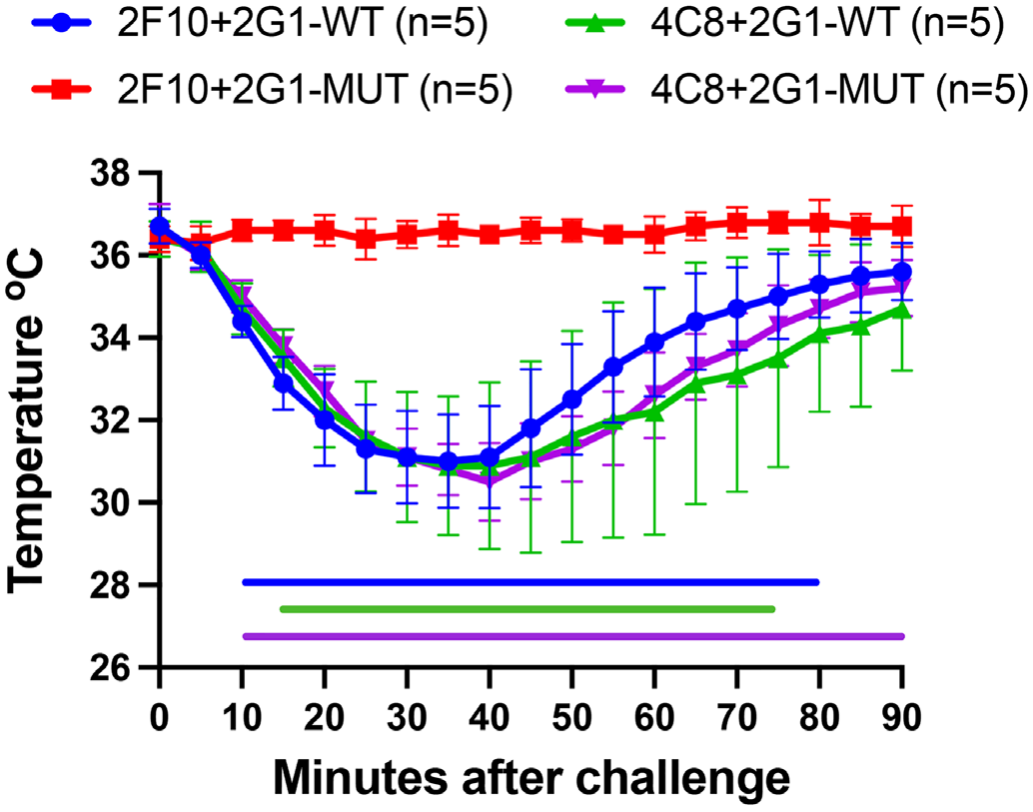
Der p 2 triple mutant (D59K, L61K, and K100D) allergen does not induce anaphylaxis in hFcεRI mice. Human FcεRIα–transgenic mice were sensitized with 100 µg total of human Der p 2-specific IgE mAbs 2F10 + 2G1 or 4C8 + 2G1. Animals were challenged with 50 µg of recombinant Der p 2 wildtype or triple mutant (D59K, L61K, and K100D). Anaphylaxis was monitored using an implanted temperature probe for 90 min following challenge. Time points with calculated *P-*values < 0.05 are underscored with a colored bar. Data are means ± SD of each experimental group. The number of mice (n) for each experimental group is shown.

A human FcεRIα–transgenic mouse model of passive systemic anaphylaxis was used to functionally test the ability of the Der p 2 triple mutant (D59K, L61K, and K100D) allergen protein to induce mast cell activation and degranulation. This model tests whether FcεRI is cross-linked by allergen and, thus whether the nonoverlapping IgE mAb pair passively transferred to mice is capable of binding the allergen. Mice were sensitized by i.v. route using purified IgE mAb pairs specific to Der p 2, 2 days prior to challenge by i.p. route with 50 µg of purified recombinant Der p 2 wildtype or mutant. Three IgE mAbs 2F10, 2G1, and 4C8 were selected because they bind to nonoverlapping epitopes (27). Anaphylaxis was measured by the decrease in body temperature using implanted temperature probes. Figure 4 shows that the IgE mAb pair 2F10 plus 2G1 and 4C8 plus 2G1 resulted in significant anaphylaxis when mice were challenged with recombinant Der p 2 wildtype protein. Mice sensitized with 4C8 plus 2G1 also exhibited significant anaphylaxis when challenged with the Der p 2 triple mutant protein. The decrease in temperature displayed by these three experimental groups reflects systemic anaphylactic reactions that could have only occurred with two mAbs’ ability to simultaneously bind Der p 2 and cross-link FcεRI. However, mice sensitized with 2F10 plus 2G1 showed no decrease in core body temperature when challenged with the Der p 2 triple mutant protein. In this case, the lack of systemic anaphylactic reactions shows that IgE mAb 2F10 did not sufficiently bind to the allergen and cross-link FcεRI. Moreover, the resulting anaphylaxis seen in mice sensitized with 4C8 plus 2G1 and challenged with the Der p 2 triple mutant protein shows that IgE binding sites on Der p 2 other than the 2F10 epitope (i.e. the 4C8 and 2G1 epitopes) are not altered and retain their conformational integrity.

## Discussion

Structure-based design of vaccine antigens, making use of naturally occurring human mAbs and atomic-level structural information, has become commonplace for the rapid development of vaccines against emerging infectious pathogens (31). These basic concepts can now be used to combat allergic diseases by aiding in the design of improved diagnostics and immunotherapeutics. For this, the identification of human IgE epitopes is a major goal of allergy research. However, this goal has been hampered by the difficulty to obtain human IgE mAbs, given the polyclonal nature and low frequency of IgE in blood (9, 10). In this study, we report a structural and immunological analysis of the interaction between a human IgE mAb (2F10) and the house dust mite allergen Der p 2, as it occurs *in vivo* (19, 20). The IgE mAb was obtained from a human hybridoma resulting from the fusion of a B cell from a mite-allergic patient and a myeloma partner, and therefore, contains the natural pairing of the heavy and light chains. This is a substantial discovery in the field of allergen–antibody research as compared to previous X-ray crystallography studies by us and others of structures of allergens in complex with either mouse IgG mAbs as surrogates for IgE (11–18), a human IgG antibody derived from a single B cell of an allergic individual (22), or IgE constructs from phage display combinatorial libraries, which do not necessarily have the natural pairing of heavy and light chains (19, 20). In addition, the immunodominance of this IgE mAb 2F10 (or an IgE mAb of overlapping specificity) versus the total IgE polyclonal response was supported by its capacity to inhibit up to 36% binding of polyclonal IgE to Der p 2. This finding showed that this particular IgE mAb recognizes one of the epitope regions involved in the polyclonal IgE response to Der p 2 from the mite-allergic subjects tested.

Comparison of allergen–antibody interfaces from 17 complexes revealed that this unique IgE mAb–allergen interface had comparable structural parameters to the other 16 allergen–antibody complexes, mostly containing IgG mAb fragments. These parameters include residues from the paratope, hydrogen bonds in heavy and light chains, hydrogen bonds involving main or side chains of antibodies, and secondary structure elements. The epitope for IgE mAb 2F10 is located close to the apical C-terminal end of Der p 2, with a surface area (∼750 Å^2^), comparable (although smaller than the average) to the area of epitopes in other allergen–antibody complexes (18). Most of the interface area (72%) corresponds to the region between the allergen and the heavy chain of the antibody, specifically the CDR3 that has been reported to be sufficient for most antibody specificities (32). IgE antibodies are produced following class switching from IgM, often via IgG antibodies, and thus beyond the observed evidence of affinity maturation versus the corresponding germline sequence, the actual nature of allergen recognition is not expected to be radically different, or in fact different at all, from other high-affinity IgG antibody/antigen or antibody/allergen complexes (what makes the antibody an IgE is the constant region of the heavy chain, not the variable domains). This is in fact what was found, namely that the antibody/allergen interface described here is typical of that seen in other antibody/allergen complexes.

It is also worth noting a possible dimerization of Der p 2 given the relative position of Der p 2 pairs in the crystals. The putative dimers identified in the 2F10–Der p 2.0103 crystal structure are almost exactly the same as the putative dimeric assemblies observed in the crystal structure of Der p 2 alone (PDB code: 1KTJ). In fact, Der p 2 is present in solution as both monomers (mostly) and dimers at high allergen concentration. However, whether this dimerization occurs *in vivo*as well, at environmental levels of exposure, is unknown. Dimerization of allergens in complexes with antibodies has been reported for other allergens, such as Bla g 2 and Bos d 5 in complex with Fabs (11, 19). The ability of Der p 2 to form dimers could potentially facilitate IgE cross-linking, through the same epitope present on each of the molecules forming the dimer. While Der p 2 dimerization is possible, superantigen-like recognition of the allergen in addition to the conventional binding by the same Fab, as reported for Phl p 7, was not observed in the 2F10–Der p 2.0103 complex (22).

The structural basis of the IgE mAb 2F10 interaction with Der p 2 led to the rational design of mutated allergens with impaired capacity to bind IgE mAb 2F10, which allowed the investigation of: (1) the loss of their IgE binding capacity, and (2) their functional ability to cross-link IgE *in vivo*using a mouse model of passive systemic anaphylaxis. First, site-directed mutagenesis analyses of the 2F10 epitope revealed three amino acids (Asp59, Leu61, and Lys100) that are important for antibody recognition. The position of these three residues is in agreement with the location of the 2F10 binding area previously predicted by NMR, which comprised residues 54 to 65, 97 to 99, 103 to 107, and 121 to 123 as likely responsible for antibody binding (33). However, information originating from the crystal structure of the Der p 2.0103–2F10 Fab narrows down the region that is most important for antibody binding and excludes residues 121 to 123 that are close to the epitope, but are not directly involved in antibody recognition. The mutations of residues Asp59, Leu61, and Lys100 were designed to affect either hydrophobic interactions (Leu61 and Lys100) or hydrogen bonds (Asp59). Amino acid substitutions at these three positions were sufficient to result in a significantly reduction (up to 2,200-fold versus the wildtype) of IgE mAb 2F10 binding to three of the mutants (#3, 4, and 5) in inhibition assays, while maintaining the correct immunoglobulin-like fold typical of wildtype Der p 2 (as proven by immunoassays, NMR, and CD spectra of the mutants). An observation from these inhibition experiments is that as the concentration of the inhibitor allergen increased to 1 µg/mL, there was an increased inhibition of 2F10 binding to Der p 2 (especially for the two mutants containing the K100D substitution). This effect could result from an increased binding between the 2F10 antibody and the inhibitor mutant, due to binding outside the mutated regions (Supplementary Material Appendix, Supplemental Results for additional details). Der p 2 is a very flexible molecule, and certain amino acid substitutions might lead to changes in the position of other residues, outside the mutated regions, which could increase the binding of 2F10 to the mutant.

A first inference from the site-directed mutagenesis analysis was that the nature of the substitution is a crucial determinant of the effect on antibody binding: substitution of residues 59 and 61 to alanines (*mutant*#1) produced a weaker reduction of IgE mAb 2F10 binding than to lysines (*mutant*#3; 12 and 2,200-fold, respectively, versus the wildtype), which are longer and positively charged residues and can better interfere with antibody recognition. Interestingly, a single mutation (K100D) in *mutant*#4 showed the same strong effect as four or two mutations in *mutants*#2 and #3, respectively, highlighting the importance of the hydrophobic interactions established by Lys100. In addition, mutation of the adjacent epitope for the IgG mAb 7A1 reduced the binding of 2F10 IgE mAb to Der p 2 *mutant*#1, with an effect comparable to the one observed for *mutants*#3, #4, and #5 (Supplementary Material Appendix, Supplemental Results for additional details). Most of the amino acid substitutions performed (except L61A) affected the complementarity at the allergen–antibody interface (e.g. Asp to Lys mutation or Lys to Asp mutation), which is a determinant of antigen–antibody interaction (34).

In agreement with the data from the other mutants, the 2F10 epitope triple mutant (D59K L61K K100D) showed a strong reduction of IgE mAb 2F10 antibody binding. This triple mutant had also a reduced capacity to inhibit polyclonal IgE antibody binding to Der p 2 compared to the wildtype allergen for the 10 subjects tested. This last result indicates that the 2F10 epitope is important, if not dominant, for the allergic response to Der p 2. In addition, a high and significant inverse correlation was found between the maximum inhibition of the polyclonal IgE binding to Der p 2 by the triple mutant and the IgE titers for the 10 subjects tested. A larger inhibition observed for lower IgE levels to Der p 2 could indicate that the response is less polyclonal at low IgE levels, and therefore, the 2F10 IgE represents a larger proportion of the polyclonal IgE response. In other words, the IgE mAb associated with the 2F10 epitope seemed to be more relevant for subjects with lower Der p 2-specific IgE levels.

The 2F10 epitope triple mutant would be expected to activate mediator release in *in vitro*standard basophil or mast cell activation tests because it is correctly folded, and contains at least two other intact IgE epitopes (the previously reported overlapping IgE mAb 5D10, 2G1, and 1B8 (27), and the recently identified IgE mAb 4C8). Therefore, this mutant was tested for its capacity to cross-link IgE antibody pairs in a mouse model of passive anaphylaxis. This model provides an *in vivo*assessment of physiological responses to the mutant upon cross-linking selected pairs of IgE mAbs. According to design, the 2F10 epitope triple mutant, as opposed to the wildtype, did not induce anaphylactic responses when the pairs used to sensitize mice were the IgE mAbs 2F10 and 2G1. In contrast, mice sensitized with 4C8 plus 2G1 exhibited significant anaphylaxis when exposed to the triple mutant or the wildtype. These results provided once more proof that the mutated allergen preserves the correct folding and functional epitopes for the IgE mAbs 4C8 and 2G1.

The route of Der p 2 exposure in humans (inhalation) differs from the intraperitoneal injection for challenging mice. The allergen doses used in the mouse model (50 μg purified recombinant Der p 2) are difficult to compare with the human allergen exposure levels, which are measured as allergen concentration in dust (µg/g) (1). Mite allergen levels for risk of sensitization and the development of asthma were proposed for Der p 1 to be 2 µg/g of dust, and the higher level of 10 µg/g was proposed as a major risk factor for the development of acute asthma in mite-allergic individuals (35). Der p 2 levels in dust oscillate between 0.1 and 100 μg/g (36), but are commonly quite low (0.2 to 1 μg/g). Despite these differences in allergen dose and route of exposure between human and mice exposures, the mouse model was appropriate and useful to prove mast cell activation *in vivo*, considering the previously reported importance of the high affinity IgE receptor (FcεRI) in mouse anaphylaxis (37, 38). The structure-based prediction of the antibodies and allergens that would be able to activate mediator release in the mouse model of passive anaphylaxis were confirmed, showing the functional relevance of the IgE mAb 2F10 *in vivo*.

The results from this study have implications for the future of immunotherapy. Currently, the administration of increasing doses of allergen extracts can lead to side-effects due to cross-linking of IgE antibodies by allergen. Hypoallergenic chemically modified extracts (allergoids), with reduced capacity to bind and cross-link IgE, have been successful for immunotherapy (39, 40). However, they are not approved by the FDA, they are not structurally well-defined molecules, and they are difficult to standardize (as extracts and unlike recombinant hypoallergens). On the other hand, the concept of recombinant hypoallergens has not been sufficiently explored. Only one of few published immunotherapy trials using recombinant allergens involved hypoaeroallergens (Bet v 1 fragments and trimer) (41, 42). In the current study, structural features of the allergen–IgE antibody interaction were revealed at high resolution. This allowed a structure-based rational design of epitope mutants with impaired IgE mAb 2F10 binding. This is a first step towards the creation of hypoallergens, for which mutations of additional IgE epitopes will need to be incorporated. By knowing the allergen–IgE interactions, such as the one described herein in detail, modified hypoallergens can be designed and produced that will reduce the risk of side effects. Interestingly, the relevance of the IgE mAb 2F10 extends to homologous allergens from other mite species, since this IgE also recognizes other house dust mite allergens from the same Pyroglyphidae family (*D. farinae* and *E. maynei*), which has been recognized as the most important source of mite allergens since 1967 (35). Modification of the epitope to reduce 2F10 binding might lead to therapeutic molecules for allergies to mites from the Pyroglyphidae family, which are very common, especially in temperate areas. The tailored selection of allergens relevant to each patient combined with structurally based hypoallergens, easier to standardize versus extracts, should allow achieving maximal dose with relatively few injections. These cocktails could be used in the future as an alternative to conventional allergen extracts currently used for immunotherapy.

## Materials and Methods

### Generation of a human hybridoma producing IgE mAb 2F10

IgE-secreting human hybridomas were generated using a methodology that was recently described (27, 29). Dust mite-allergic subjects were recruited from within the Vanderbilt University Medical Center (VUMC). The prevalence of certain species of pyroglyphid dust mites in this area, specifically in Memphis, TN, has been reported to be 93.5% for *D. farinae*, 77.4% for *D. pteronyssinus*, 12.9% for *E. maynei*, and 3.2% for *Blomia tropicalis* (43). Diagnosis and recruitment details, as well as the human hybridoma generation and determination of antibody binding affinity for Der p 2, are described in Supplementary Material Appendix (Supplemental Material and Methods).

### Antibody sequencing of IgE antibodies expressed by hybridoma cell lines

RNA extracted from hybridoma cells was reverse transcribed to cDNA, which was used to sequence the variable regions of the heavy and light chains of the antibodies by Rapid Amplification of cDNA Ends (RACE) as described in Supplementary Material Appendix (Supplemental Material and Methods).

### Expression of 2F10 Fab and IgE–IgG antibody constructs

The DNA encoding for antibody constructs (2F10 Fab, 2F10 IgE–IgG, 1B8 IgE–IgG, and 2G1 IgE–IgG) was synthesized by GeneArt (Regensburg, Germany) with codon optimization for expression in CHO cells. IgE–IgG constructs are hybrid monoclonal antibodies that comprise the IgE mAb Fabs (containing the paratope) and the Fc from an IgG as described in Supplementary Material Appendix (Supplemental Material and Methods). The synthesized DNA fragments containing either the light chain or heavy chain were cloned separately into the pcDNA3.4 vector (Invitrogen, San Diego, CA, USA). Transfection grade plasmids were purified, quantified, filtered, and transfected in a 1:1 ratio of heavy to light chains (combined with ExpiFectimine) into ExpiCHO-S cells (Thermo Fisher, Waltham, MA, USA). Full description of culturing conditions and purification of antibody constructs is available in Supplementary Material Appendix (Supplemental Material and Methods).

### X-ray crystallography of the Der p 2–IgE mAb 2F10 Fab complex

Recombinant Der p 2.0103 and 2F10 Fab were mixed at 1:1 molar ratio to prepare a complex that was purified by size exclusion chromatography. The purified complex was used for crystallization experiments by vapor diffusion. Data collection was performed at the Advanced Photon Source, Argonne National Laboratory (Lemont, IL) at 100 K. Full crystallization details and computational approaches are available in Supplementary Material Appendix (Supplemental Material and Methods).

### Production of wildtype and mutated Der p 2 allergens

Natural Der p 2 was purified from *D. pteronyssinus* spent mite cultures. Amino acid substitutions in the epitope were performed by site-directed mutagenesis. Recombinant allergens were expressed and purified, and their correct folding was assessed by NMR, CD spectra, and immunoassays. Methodological details are in Supplementary Material Appendix (Supplemental Material and Methods).

### Immunoassays to assess the relevance of IgE mAb 2F10 and to measure antibody binding to mutants

To assess the relevance of the IgE mAb 2F10, inhibition assays of human polyclonal IgE antibody binding to Der p 2 were performed using chimeric IgE–IgG constructs of 2F10 as inhibitor. Other chimeric IgE–IgG antibodies (2G1 and 1B8, both binding to overlapping epitopes at the opposite end of the 2F10 binding site) were also used for comparison. As a control, inhibition of IgE mAb 2F10 to Der p 2 by IgE–IgG 2F10 was also performed.

To assess the effects of site-directed mutagenesis on antibody binding to the mutants two kinds of assays were performed: (1) direct binding of monoclonal antibodies to Der p 2 (wildtype and mutants) coating the plates, and (2) inhibition assays in which allergens (wildtype or mutants) were preincubated with IgE mAb or polyclonal antibodies before measuring antibody binding to the wildtype allergen coating the plate. Full details of the experiments are available in Supplementary Material Appendix (Supplemental Material and Methods).

### Assessment of reactivity of the 2F10 epitope triple mutant in a mouse model of passive anaphylaxis

Mouse studies were carried out in accordance with recommendations in the Guide for the Care and Use of Laboratory Animals of the National Institutes of Health. Human FcεRI–transgenic mice [B6.Cg-Fcεr1a^tm1Knt^Tg(FCER1A)1Bhk/J] were purchased from The Jackson Laboratory (stock 010506), bred, and genotyped. These mice carry two gene mutations: the human Fc fragment of IgE receptor α polypeptide (FCER1A) under control of the human FCER1A promoter and a mutation targeting Fcεr1a^tm1Knt^, blocking expression of murine FCER1A. Human IgE can induce anaphylaxis in mice hemizygous for the transgene and homozygous for targeted deletion of mouse FcεRI. Transgenic mice were sensitized by i.v. injection of 100 μg total IgE and challenged by i.p. injection of 50 μg purified recombinant allergen. Changes in mouse core body temperature were monitored over 90 min using implanted temperature probes. Statistical analysis is described in Supplementary Material Appendix (Supplemental Materials and Methods).

## Supplementary Material

pgac054_Supplemental_File

## Data Availability

The final model of the Der p 2.0103–2F10 Fab complex together with structure factors were deposited to the PDB with accession code 7MLH.
