# Correction to: Human IgE monoclonal antibody recognition of mite allergen Der p 2 defines structural basis of an epitope for IgE cross-linking and anaphylaxis *in vivo*

**DOI:** 10.1093/pnasnexus/pgad459

**Published:** 2024-01-30

This is a correction to: Kriti Khatri, Crystal M Richardson, Jill Glesner, Anyway Brenda Kapingidza, Geoffrey A Mueller, Jian Zhang, Cole Dolamore, Lisa D Vailes, Sabina Wünschmann, R Stokes Peebles Jr, Martin D Chapman, Scott A Smith, Maksymilian Chruszcz, Anna Pomés, Human IgE monoclonal antibody recognition of mite allergen Der p 2 defines structural basis of an epitope for IgE cross-linking and anaphylaxis *in vivo*, *PNAS Nexus*, Volume 1, Issue 3, July 2022, pgac054, https://doi.org/10.1093/pnasnexus/pgac054

During a retroactive audit conducted by *PNAS Nexus*, it was discovered that this paper was missing a statement acknowledging compliance with the *PNAS Nexus* Human and Animal Participants and Clinical Trials policy:

The full study protocol involving human participants was approved by the VUMC Institutional Review Board and informed consent was obtained from all participants. The Institutional Animal Care & Use Committee at VUMC approved the animal studies protocol (approval number M1700156).

This error has been corrected in the original article.

