# Supplementary material for: Human IgE monoclonal antibody recognition of mite allergen Der p 2 defines structural basis of an epitope for IgE cross-linking and anaphylaxis in vivo
Source: PNAS Nexus. 2022 Jun 2;1(3):pgac054. doi: 10.1093/pnasnexus/pgac054 (PMC9248284; doi:10.1093/pnasnexus/pgac054)
Supplement: pgac054_Supplemental_File [file pgac054_supplemental_file.docx]

**
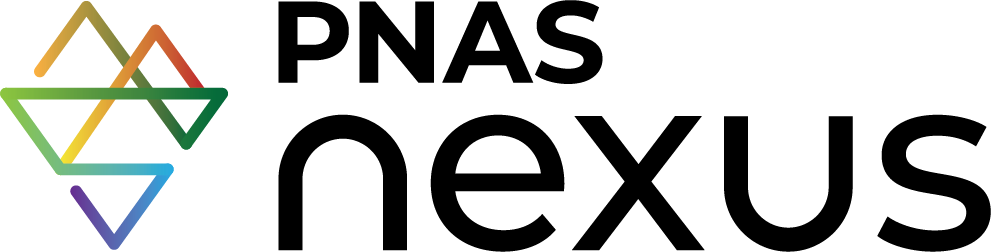
**

**Supplementary Information for**

Human IgE monoclonal antibody recognition of mite allergen Der p 2 defines structural basis of an epitope for IgE cross-linking and anaphylaxis *in vivo*

Kriti Khatri^a1^, Crystal M. Richardson^b1^, Jill Glesner^b^, Anyway Brenda Kapingidza^a,c^, Geoffrey A. Mueller^d^, Jian Zhang ^e^, Cole Dolamore^a^, Lisa D. Vailes^b^, Sabina Wünschmann^b^, R. Stokes Peebles Jr.^e^, Martin D. Chapman^b^, Scott A. Smith^e^, Maksymilian Chruszcz^a^, Anna Pomés^b*^

* Anna Pomés

Email: apomes@inbio.com

**This PDF file includes:**

Supplementary text

Figures S1 to S11

Tables S1 to S4

SI References

Supplementary Information Text

**Supplementary Material and Methods**

**Generation of a human hybridoma producing IgE mAb 2F10**

Dust mite allergic subjects that provided B cells were recruited from within the Vanderbilt University Medical Center (VUMC). The protocol for recruiting and collecting blood samples from allergic subjects was approved by the VUMC Institutional Review Board (IRB 141330 and 142030). Diagnosis was based on clinical history and presence of IgE Ab to dust mite, specifically *Dermatophagoides farinae* and/or *D. pteronyssinus*. Der p 2-specific IgE antibody (Ab) levels were measured in human sera by using ImmunoCAPs (d203) in a ImmunoCAP system (Phadia™ 250 Immunoassay Analyzer; Thermo Fisher Scientific, Portage, MI). Peripheral blood mononuclear cells (PBMC) were isolated from a blood donor who had positive skin prick test for dust mite *D. farinae* (15x10 mm wheal) and dust mite *D. pteronyssinus* (10x8 mm wheal) as well as food allergies. B cells were grown in 96 well tissue culture plates for 7 days in the presence of γ-irradiated NIH3T3 fibroblast line genetically engineered to express cell-surface human CD154 (CD40 ligand), secreted human B cell activating factor (BAFF) and human IL-21 (kindly provided by Dr. Deepta Bhattacharya; University of Arizona). B cell receptors are globally cross-linked with a mixture of murine anti-human light chain mAbs, while memory B cell activation and expansion is improved with addition of TLR9 stimulation by CpG oligodeoxynucleotides (1, 2). IgE antibody secretion was determined by ELISA as previously described (3, 4). Cells within wells containing IgE were fused to a myeloma partner (HMMA2.5 nonsecreting myeloma cells) by electrical cytofusion. Human hybridomas were then selected in hypoxanthine-aminopterin-thymidine (HAT) medium and biologically cloned using flow cytometric single cell sorting. Finally, IgE secreting hybridomas were grown in serum free medium before IgE mAb were purified by Omalizumab chromatography. The Der p 2-specificity of IgE mAb 2F10 (that did not bind Der p 1) was proven by immunoassays.

**Affinity-related IgE mAb 2F10 analyses**

Regarding affinity maturation, IMGT/V-QUEST analyses were performed for the heavy and light chains of the IgE mAb 2F10, which were found to share 94.0% and 95.7% identity, respectively, with the germline sequence (**Table S2**). These can be considered as sequences moderately mutated versus the germline if we consider (there is no consensus): near germline (>95%), moderate (<95 to >85%), heavily (<85%), although there are not many IgE sequences available to make statements about relative mutation frequencies.

To determine the affinity (K_D_) of the IgE mAb 2F10, scouting analysis and full kinetics binding assays were performed using surface plasmon resonance (SPR) on a Biacore 3000 at 25°C, at Precision Antibody in Columbia, Maryland, USA. Flow cells on the CM5 chip were coated with mAb 2F10. Coating was done by using EDC-NHS amine coupling method as per GE manufacturer’s instructions. Unoccupied sites were blocked with methanol amine. Binding of Der p 2 antigen to the mAb 2F10 was monitored in real time. Scouting was done using 200 nM of Der p 2 and a full kinetics experiment was performed by adding a range of Der p 2 in 2-fold serial dilutions to the chip-containing chamber, from lowest to highest concentration. Although Biacore scouting analysis allows for relative antibody ranking based on binding kinetics, full kinetics binding affinity measurements are more accurate for determination of K_D_. The K_D_ was determined from the observed k_on_ and k_off_ values. Steady state kinetics was used to determine K_D_ for any interactions with a fast off rate.

**Antibody sequencing of IgE antibodies expressed by hybridoma cell lines**

Hybridoma cell line expressing human IgE mAb 2F10, 1B8, 2G1 or 4C8 were cultured in Medium E (Stem Cell Technologies, Vancouver, BC, Canada). Cell culture was maintained at 37°C, 5% CO_2_. Pellets were stored at -80°C until RNA extraction. Cells were lysed with the QIAshredder spin column (Qiagen, Germantown, MD, USA). Total RNA extraction was performed with RNeasy Mini Kit (Qiagen, Germantown, MD, USA). Reverse transcription was used to synthesize cDNA from total RNA. The variable region of the IgE mAb was sequenced by Rapid Amplification of cDNA ends (RACE) using a SMARTer RACE 5’/3’ kit (Takara, San Jose, CA, USA). The 5’ RACE reaction was done with primers designed from the constant antibody region. Separate PCR reactions were done for heavy and light chains of antibodies. Following PCR, cloning of the DNA encoding each antibody chain into a pRACE vector was done with In-Fusion HD Cloning Kit (Takara, San Jose, CA, USA). DNA was isolated by plasmid preparation with Qiagen MiniPrep Kit (Germantown, MD, USA) and sequenced with M13F and M13R primers (Eurofins Genomics, Louisville, KY, USA). The antibody sequences were confirmed by alignment of forward and reverse sequencing of two separate colonies performed in duplicate.

**Expression of 2F10 Fab and IgE-IgG antibody constructs**

The DNA encoding for antibody constructs (2F10 Fab, 2F10 IgE-IgG, 1B8 IgE-IgG, and 2G1 IgE-IgG) was synthesized by GeneArt (Regensburg, Germany) with codon optimization for expression in CHO cells. The synthesized DNA fragments containing either the light chain or heavy chain were cloned separately into the pcDNA3.4 vector (Invitrogen, San Diego, CA, USA). Transfection grade plasmid purification was performed with a PureLink MidiPrep Kit (Life Technologies, Carlsbad, CA, USA). Plasmids were quantified by UV spectroscopy (260 nm) and DNA was sterilized by filtration through a 0.2 µm spin filter (Corning, Tewksbury, MA, USA). Plasmids were transfected in a 1:1 ratio of heavy to light chains into ExpiCHO-S^TM^ cells (Thermo Fisher, Waltham, MA, USA) by combining the DNA with ExpiFectimine^TM^ (Thermo Fisher, Waltham, MA) for complexation reaction. Cells were cultured for 13 days in ExpiCHO media (Thermo Fisher, Waltham, MA, USA) at 37°C, 8% CO_2_ for secretion of each of the recombinant antibodies. The cell supernatant was collected, centrifuged at 2,000 x g for 30 minutes, and filtered through a 0.2 µm vacuum filter (Corning, Tewksbury, MA, USA). Affinity purification of 2F10 Fab was performed using a HisTrap^TM^ HP prepacked column (Cytivia, Marlborough, MA, USA) according to manufacturer’s instructions. Purification of the chimeric antibodies (2F10 IgE-IgG, 1B8 IgE-IgG, and 2G1 IgE-IgG) was performed by affinity chromatography using a Protein G column (Cytivia, Marlborough, MA, USA).

**Preparation of the Der p 2-IgE mAb 2F10 Fab complex and crystallization**

Der p 2.0103 and 2F10 Fab were mixed at 1:1 molar ratio to prepare a complex that was purified by size exclusion chromatography. The purified complex was used for crystallization experiments by vapor diffusion. Specifically, 2F10 Fab was concentrated to 12.9 mg/mL and mixed with Der p 2.0103 (1.3 mg/mL) in 1:1 molar ratio. The solution was incubated overnight on ice and passed through a size exclusion column (HiLoad Superdex 200) equilibrated with buffer, 20 mM sodium phosphate, 150 mM NaCl and 250 mM imidazole at pH 7.4. Fractions containing Der p 2.0103-2F10 Fab complex were pooled and concentrated to 4 mg/mL. This solution was used for crystallization experiments, which were performed using vapor diffusion method at 293K. The best diffracting crystals were obtained from Index screen (Hampton Research) and conditions containing 0.1 M sodium acetate and 2.0 M ammonium sulfate at pH 4.5 or 0.1 M citric acid and 2.0 M ammonium sulphate at pH 3.5. The structure described in this manuscript was obtained from crystals growing from conditions containing citrate. Prior to data collection, the crystals were cryo-cooled in liquid nitrogen without addition of cryo-protectants.

**Data collection, structure determination and model validation**

Data collection was performed at the Southeast Regional Collaborative Access Team 22-ID at the Photon Advanced Source, Argonne National Laboratory (Lemont, IL) at 100K. Diffraction images were processed using HKL-2000 (5), and data collection statistics are summarized in **Table S1**. Structure solution was performed using molecular replacement with MOLREP (6) incorporated into HKL-3000 (7), which also uses various programs from the CCP4 package (8). PDB structure 1KTJ (Der p 2) and 6OY4 (Fab) were used as search models. The initial structure was rebuilt with COOT (9), and refined with COOT, REFMAC (10) and HKL-3000. COOT, HKL-3000 and MOLPROBITY (11) were used for structure validation. The final model of the Der p 2.0103-2F10 Fab complex together with structure factors were deposited to the PDB with accession code 7MLH. The structure of the complex was used as the base for the design of Der p 2.0103 mutants.

**Various computational approaches**

Structural images were created with Pymol (12). The 2F10 – Der p 2.0103 interface was analyzed using PDBePISA (13). Surface complementarity was calculated using CCP4 package and SC program that is based on an approach developed by Lawrence and Coleman (14).

**Production of natural Der p 2 and recombinant wildtype and 2F10 epitope mutants**

Natural Der p 2 (nDer p 2) was purified from *D. pteronyssinus* spent mite cultures, using benzamidine affinity chromatography for removal of serine proteases followed by mAb 7A1 affinity chromatography. Purity was >95% by silver stained SDS-PAGE.

Recombinant house dust mite allergens Der p 2.0103 and Der p f 2.0103 were expressed in *Pichia pastoris* and purified via mAb affinity chromatography (15). The Der p 2.0103 variant shares the same sequence as the most frequent variant Der p 2.0101, except for three additional substitutions, one of which confers capacity to bind the IgG mAb 1D8. Recombinant Der p 2.0101 was expressed in *P. pastoris* with a C-terminal 6xHis-tag and purified via immobilized metal affinity chromatography (IMAC) followed by gel-filtration chromatography. Recombinant storage mite protein Gly d 2.0101 was expressed in *P. pastoris* with a C-terminal 6xHis-tag and purified using IMAC. Recombinant Lep d 2.0101 was expressed in *P. pastoris* and recombinant Eur m 2.0101 in *E. coli*, both were purified via multi-step ultrafiltration. Purity of ~95% was assessed on silver-stained SDS-PAGE.

Site-directed mutagenesis of the IgE mAb 2F10 epitope on Der p 2.0103 was performed using the QuikChange Site-Directed Mutagenesis kit (Agilent Technologies, Santa Clara, CA). DNA encoding for wildtype Der p 2.0103 inserted in the pPICZαA vector was used as template for mutagenesis. The sequences of the mutated DNA were confirmed before linearization and transformation into the *P. pastoris* strain KM71. The 2F10 epitope Der p 2 mutants were expressed by methanol induction and were purified by specific-antibody affinity chromatography using the mAb 1D8, as previously described (15).

**Assessing folding of wildtype Der p 2 and mutants**

Folding of the five mutants versus wildtype Der p 2.0103 was assessed in three ways. First, protein folding was tested by ^1^H-NMR using watergate-3919 water suppression. Each nonlabelled mutant (228-269 µg) and wildtype allergen (750 µg) was examined in phosphate buffered saline (PBS) buffer, 10% deuterated water (D_2_O), and 0.02 mM 4,4-dimethyl-4-silapentane-1-sulfonic acid (DSS) was added for referencing. To compare with an unfolded state, wildtype Der p 2 was diluted 1:1 with 8 M urea and 2 mM DTT for a final concentration of 4 M urea and 1 mM DTT. 1D NMR spectra were acquired with urea and/or water suppression as needed with an Agilent DD2 600MHz console. Second, 150 µL of the same samples used for the NMR measurements were examined in a JASCO CD spectrometer using a 1 mm cuvette. Finally, direct binding immunoassays assessed if the overall fold of Der p 2 was conserved in the wildtype and the mutants by testing their ability to bind mAbs 1D8 and α-DpX, antibodies whose respective epitopes lie distal from that of mAb 2F10 (4).

**Inhibition of polyclonal IgE antibody binding to Der p 2 by 2F10 IgE-IgG**

Plasma from dust mite allergic patients (n = 10) used in IgE inhibition assays were acquired from PlasmaLab International (Everett, WA) which operates in full compliance of Food and Drug Administration regulations. Informed donor consent was obtained from each individual prior to the first donation. Der p 2 IgE antibody titers were measured by a Thermo Fisher Scientific ImmunoCAP system (Phadia™ 250 Immunoassay Analyzer, Thermo Fisher Scientific, Portage, MI; d203 ImmunoCAPs).

A microplate was coated overnight at 4°C with nDer p 2 at 10 µg/mL. The chimeric IgE-IgG mAbs 2F10, 2G1 and 1B8, and the murine mAb αDpX were each mixed at 10 and 100 µg/mL with plasma diluted either 1:2 or 1:4 in microcentrifuge tubes while the plate was being blocked with phosphate buffered saline, pH 7.4, containing 0.05% Tween 20 and 1% bovine serum albumin (PBS-T 1% BSA) for 1 h at room temperature. The mix of antibody and plasma was then added to the microplate wells and incubated for 3 h. A 1 h incubation with mouse anti-human IgE Fc-HRP (1:1,000) (Southern Biotech, Birmingham, AL) followed, and the plate was developed using 2,2'-azino-bis(3-ethylbenzothiazoline-6-sulphonic acid) (ABTS) in 70 mM citrate phosphate buffer, pH 4.2 and 1:1,000 dilution of H_2_O_2_. Absorbance was read at 405 nm on a Bio-Tek EL800 Microplate Reader (Bio-Tek Instruments, Inc., Winooski, VT). In this and other immunoassays described below, plates were washed at room temperature three times between incubation steps with phosphate buffered saline, pH 7.4, containing 0.05% Tween 20 (PBS-T).

**Inhibition of IgE mAb 2F10 binding to Der p 2 by 2F10 antibody constructs**

The capacity to inhibit IgE mAb 2F10 binding to Der p 2 by constructs of the same epitope specificity was tested by inhibition assays using 2F10 antibody constructs (IgE-IgG, IgE or IgE Fab). IgG mAb (anti-Der p 1 4C1 or anti-Der p 2 1D8) were used for comparison. A microplate was coated overnight at 4°C with nDer p 2 at 10 µg/mL. The plate was washed with PBS-T, and then blocked with PBS-T 1% BSA. The chimeric IgE-IgG mAb 2F10 and murine IgG mAb 1D8 were each mixed at 0.1, 1, 10 and 100 µg/mL with biotinylated human mAb IgE 2F10 (B-2F10) (1:50,000), added to the microplate wells and incubated for 3 h. Similar conditions were used for 2F10 IgE, 2F10 IgE Fab and 4C1 IgG mAb. A 1 h incubation of streptavidin peroxidase (1:1,000) (Sigma-Aldrich, St Louis, MO) followed and the plate was developed as described above.

**Effect of mutations on IgE mAb 2F10 binding:**

**1. Direct binding to allergen coating the plates**

Binding of human IgE 2F10 (and the control murine IgG 1D8 and α-DpX mAb) to Der p 2 was assessed using Ab binding immunoassays. A microplate was coated overnight with rDer p 2.0103 wildtype or mutants at 10 µg/mL, which were diluted 1:2 across the plate in 50 mM carbonate/bicarbonate buffer, pH 9.6. The plate was washed with PBS-T, and blocked with PBS-T 1% BSA. Biotinylated murine IgG mAb or biotinylated human IgE mAbs (1:1,000) or non-biotinylated murine IgG mAb αDpX (1:1,000) were added. For the wells with biotinylated antibodies, addition of Streptavidin Peroxidase (1:1,000) (Sigma-Aldrich, St. Louis, MO) followed. In the case of αDpX, peroxidase-conjugated AffiniPure F(ab’)_2_ fragment goat anti-mouse IgG (1:1,000) (Jackson ImmunoResearch Laboratories, Inc., West Grove, PA) was used. The plate was developed as described above.

**2. Inhibition of IgE mAb 2F10 binding to Der p 2 by wildtype Der p 2 and epitope mutants**

A microplate was coated overnight at 4°C with rDer p 2.0103 at 10 µg/mL. B-2F10 (1:50,000) was incubated with Der p 2.0103 wildtype or mutants, each at 0.0001, 0.001, 0.01, 0.1, 1, 10 and 100 µg/mL in microcentrifuge tubes for 1 h while the plate was being blocked with PBS-T 1% BSA at room temperature. Each mix of biotinylated IgE mAb and allergen was then added to the microplate wells and incubated for 3 h. A 1 h incubation with Streptavidin Peroxidase (1:1,000) (Sigma-Aldrich, St. Louis, MO) followed, and the plate was developed as described above.

**Effect of mutations on polyclonal IgE antibody binding: Inhibition of polyclonal IgE binding to Der p 2 by wildtype and the epitope triple mutant**

A microplate was coated overnight at 4°C with rDer p 2.0103 at 10 µg/mL. The Der p 2.0103 wildtype and the triple mutant rDer p 2 D59K L61K K100D were each incubated at 0.001, 0.01, 0.1, 1, 10, 100 µg/mL with plasma at either 1:2 or 1:4 in microcentrifuge tubes for 1 h while the plate was blocked with PBS-T 1% BSA at room temperature. The mix of allergen and plasma was then added to the microplate wells and incubated for 3 h. A 1 h incubation of mouse anti-human IgE Fc-HRP (1:1,000) (Southern Biotech, Birmingham, AL) followed and the plate was developed as described above.

**Statistical analysis**

Statistical analysis of results from the mouse model of passive anaphylaxis was performed using Prism v9.2 (GraphPad) software. The comparison of temperature-change curves was performed independently for each allergen challenge and at each time point using 2-way ANOVA. Time points with calculated P values less than 0.05 were labeled and considered significant. Error bars for mouse temperature measurements represent standard deviation (SD).

**Supplementary Results**

**X-ray crystal structure of Der p 2 in complex with 2F10 IgE mAb Fab**

The IgE mAb 2F10 Fab in complex with the Der p 2.0103 variant was crystallized in P2_1_ space groups with two antibody-allergen complexes in the asymmetric unit (**Fig. 1A, B; Table S1**). The structure was determined at 2.1 Å resolution. All residues corresponding to the mature form of Der p 2.0103 and the 2F10 light chains are present in the model, whereas some regions of the heavy chain constant domains (18 residues in total) are disordered and were not included in the final model. Der p 2.0103 molecules in both complexes adopt almost exactly the same main chain conformation (rmsd 0.2 Å), and only some side chains conformations are different. Similarly, conformations of the light or heavy chains are quite similar with rmsd values 0.5 Å and 0.6 Å, respectively. The complex I is formed by chains B (Der p 2.0103), D (light chain) and E (heavy chain), while Complex II is formed by chain F (Der p 2.0103), A (light chain) and C (heavy chain).

The four residues from the IgE mAb 2F10 that resulted from mutations of the germline antibody sequence and formed hydrogen bonds are: 1) Gly101 from the heavy chain (forms a hydrogen bond through the mainchain amide group with the mainchain oxygen of Der p 2 Lys100, and 2) Tyr102 from the heavy chain (forms a hydrogen bond through the sidechain hydroxyl group with the mainchain nitrogen and hydrogen atoms of Der p 2 Lys100, 3) His92 from the light chain (forms a hydrogen bond through its mainchain carbonyl oxygen atom with the amide group of Asn103 from Der p 2), and 4) His94 from the light chain (forms a hydrogen bond through its mainchain nitrogen and hydrogen atoms with the oxygen atom from the main chain carbonyl group of Der p 2 Gly60).

**Overlap between IgE mAb 2F10 and IgG mAb 7A1**

The 2F10 epitope is adjacent to the binding site of the murine IgG mAb 7A1, whose structure we previously determined, but sufficiently separated so this antibody pair recognizes the allergen in a two-site ELISA (15). Der p 2.0103 residues Ile97 and Pro99 in both cases participate in hydrophobic interactions between the allergen and the IgE mAb 2F10 and IgG mAb 7A1. Out of these two residues, Ile97 is ~3.4-4.0 Å from the 7A1 paratope, and slightly closer to the 2F10 paratope. In the case of the Ile97 residue, it seems that changing the conformation of 1-2 residues on each antibody is enough to avoid any steric clashes. In the case of Pro99, to eliminate potential steric clashes there has to be some rearrangement of the 2F10 H-CDR1 main chain conformation. Interestingly, the 2F10 H-CDR1 does not seem to participate in Der p 2 binding, which provides an explanation for why both antibodies can simultaneously interact with Der p 2. NMR studies suggested that Der p 2.0103 residues Val63, Ile97, Gly98 and Pro99 belong to the overlapping region of the 2F10 and 7A1 epitopes (4). Our results indicate that this is true for the 97-99 fragment, but not for Val63. Interestingly, comparison of the crystal structures of the Fab with and without allergen provides information on the epitope location, while NMR chemical shifts (such as the one corresponding to Val63) show conformational changes upon antibody binding not necessarily occurring in the epitope. Namely, the conformation of the Der p 2.0103 fragment containing Val63 is different depending on the antibody that binds the allergen. In the complex with 2F10, the conformation of this Der p 2.0103 fragment is similar to the one observed in a crystal form of allergen alone, suggesting that Der p 2 does not go through major conformational changes upon 2F10 binding.

*Mutant #2* was designed to have a combination of mutations affecting these two adjacent epitopes: mutations D59A L61A in the 2F10 epitope and mutations K96E-I97E (previously reported to abolish IgG mAb 7A1 binding) (15). Interestingly, the mutation of one epitope (for IgG mAb 7A1) reduced the binding of 2F10 to the adjacent epitope, with an effect comparable to the one observed for *mutants #3*, *4* and *5*. A possible explanation for this observation is that one of the substitutions (I97E) affects an amino acid involved in binding of both antibodies 7A1 and 2F10. In fact, Ile97 and Pro99 interact with Phe55 of the IgE mAb 2F10. As mentioned above, residues 97-99 had been predicted by NMR to be in overlapping areas of the 7A1 and 2F10 epitopes (4). Another possibility is that the two mutated residues Lys96 and Ile97 from the 7A1 epitope are also important for 2F10 interaction, and by changing them, the conformation of the loop 97-104 that is important for 2F10 binding also changes, therefore affecting the IgE mAb 2F10 binding.

**Structural comparison of the 2F10-Der p 2 complex with 16 other allergen-antibody complexes**

The number of residues in the 2F10 paratope from the heavy (14) and light (6) chains were within the same range as the ones found in 16 other allergen-antibody complexes analyzed (11-18 and 5-14, respectively). The hydrogen bonds with the heavy (7) and light (4) chains were also within the same range (4-12 and 1-6 in 16 complexes, respectively). Similarly, the hydrogen bonds with the side (6) and main (5) chains were in the same range (4-12 and 0-7 in 16 complexes, respectively). The Der p 2.0103-2F10 interface has a similar number of hydrogen bonds (11) to that of the average hydrogen bonds in the other 16 allergen-antibody interfaces examined (11.4). Nevertheless, this number is greater compared with the interface between mAb 7A1 and Der p 2.0103 (7) (**Fig. 2C, D**). On average, most of the allergen-antibody interactions occurred with strands and turns (29% -range 0-67%- and 28% -range 0-63%-, respectively for the other 16 complexes; 33 and 40% for 2F10) for the 17 complexes (**Fig. S1**). No significant differences were observed between the secondary structure elements in the epitopes on Der p 2 recognized by IgE (PDB code: 7MLH) and the IgG mAb 7A1 (PDB code: 6OY4) (**Fig. S1**).

**Folding of wildtype and mutants**

The urea treated Der p 2 showed the typical narrowing of amide resonances to ~8ppm, and methyl resonances to ~0.8 ppm characteristic of unfolded proteins (**Fig. S7**, top panels). All the mutants showed similar spectra that indicates that their protein fold was maintained (**Fig. S7**, bottom panels). They all displayed similar dispersion to the wildtype and none of the mutants appear unfolded as compared to the urea treated wildtype Der p 2.

The CD spectra for wildtype and the mutants are characteristic of a largely beta sheet protein with a single broad rounded minima around 215 nm (**Fig. S8**). These are in contrast to the urea treated sample, with a sharp minimum closer to 210 nm common to unfolded proteins.

**Inhibition of 2F10 mAb binding to Der p 2 by the mutants and Der p 2 flexibility**

Figure 3B displays the inhibition of 2F10 mAb to Der p 2 by the mutants. As the concentration of the allergen inhibitors increases to 1 µg/mL, an increased inhibition of 2F10 binding to Der p 2 was observed, most likely due to an increased binding between the 2F10 antibody and the inhibitor mutant. This effect was more pronounced for the two mutants that contained the K100D substitution. One of the consequences of the K100D mutation may be formation of additional hydrogen bonds between Der p 2 and the heavy chain of the antibody. It is likely that in this mutant the Asp side chain is close enough to form hydrogen bond(s) with Gly101 and/or with the hydroxyl group of Tyr102 from the heavy chain.

Similarly, the mutation L61A may allow the side chain of His94 (antibody light chain) to form a hydrogen bond with main chain of the allergen Gly60, while L61A mutation may allow residues Ile52 and Leu99 (antibody heavy chain) to form larger hydrophobic contact with the allergen. Mutations D59K and L61K may also lead to a displacement of water molecules that are in the epitope-paratope for the wildtype protein, and form some direct interactions with the antibody.

The flexibility of the Der p 2 molecule (15) could contribute to changes in the position of certain amino acids, outside the mutated regions, that could increase the binding of 2F10 to the mutant.

**IgE mAb 2F10 is a major contributor to the polyclonal IgE response to Der p 2**

To establish the degree of biological relevance of the human IgE mAb 2F10, based on its contribution to polyclonal IgE responses to Der p 2, its ability to inhibit binding of IgE to Der p 2 in plasma obtained from allergic subjects who were highly sensitized to Der p 2 was investigated. This was performed by using hybrid antibodies as inhibitors. The hybrid mAb 2F10 (2F10 IgE-IgG) comprises the Fab of the IgE mAb 2F10 (containing the paratope) and the Fc from an IgG, and its capacity to inhibit binding of biotinylated 2F10 to Der p 2 was previously confirmed. Other inhibitors tested were two other IgE mAb hybrids (2G1 and 1B8 IgE-IgG) and the murine IgG mAb α-DpX (which all bind to overlapping epitopes on the opposite side of the 2F10 epitope on Der p 2). Each of these molecules was predicted to inhibit other clones involved in the polyclonal IgE response.

Plasma specimens used in the inhibition assay contained very high levels of Der p 2-specific antibodies (specific IgE class 4; mean 41.8 ± 15.6 kU_A_/L; range 24.0-65.7 kU_A_/L; n=10). The individual IgE titers for the 10 plasma are shown in **Fig. S11**. Of all the three hybrid IgE-IgG mAb inhibitors tested, the 2F10 antibody showed the largest inhibition in 9 out of 10 specimens (21.4% mean inhibition; range 11.9-36.1%; n = 10), whereas 2G1 and 1B8 inhibited IgE binding by 10.1% (range 1.2-19.5) and 12.7% (range 3.7-21.8), respectively (**Fig. S9**). The murine IgG mAb α-DpX, which binds an epitope that overlaps with epitopes for IgE mAb 2G1 and 1B8, inhibited the most in 6 out of 10 plasma when compared with all of the IgE mAb, with a mean inhibition of 21.1% (range 12.1-34.3 n = 10) similar to results for IgE mAb 2F10 (**Fig. S9**). These results indicate that IgE mAb 2F10 corresponds to an IgE clone that is a major contributor to the polyclonal IgE response to Der p 2, and that additional IgE clones that are distinct from the one associated with 2F10, also contribute to the response.

**Structural basis for the recognition of other mite allergens by 2F10 IgE mAb**

As the human IgE mAb 2F10 recognizes Der p 2.0103 residues 58-64 and 97-103, the sequences of the corresponding fragments in homologous allergens from house dust mite (Der p 2.0101, Der p 2.0103, Der f 2 and Eur m 2) and storage mites (Lep d 2 and Gly d 2) listed in the WHO/IUIS Allergen Nomenclature database ([www.allergen.org](http://www.allergen.org)) were analyzed. The sequence analysis (**Fig. S4**) together with the experimental model of the 2F10 Fab-Der p 2.0103 structure suggests that 2F10 should be able to bind all Group 2 house dust mite allergen variants that are currently officially registered in the database. The residues that are different in the corresponding 2F10 epitope of all the analyzed Der p 2 variants should not prevent 2F10 binding, and all major interactions listed above should be preserved. For example, in the epitope fragment composed of residues 97-102, the amino acids that are different among Group 2 isoallergens (**Fig. S4A**) are mainly involved in interactions through their main chains. A similar situation is observed for the more sequence diversified fragment 58-64. Here all Der p 2 isoallergens have the same sequence, while the differences observed for the corresponding fragments of Der f 2 and Eur m 2 represent conservative amino acids substitutions, which should preserve the pattern of interactions observed for the 2F10-Der p 2.0103 complex. In contrast, only Pro99 is conserved in the two corresponding areas of the epitope for the allergens from storage mites (**Fig. S4B**). Accordingly, the IgE mAb 2F10 bound to recombinant dust mite allergens Der p 2.0101, Der p 2.0103, Der f 2.0103 and Eur m 2.0101, but not to Gly d 2.0101 and Lep d 2.0101 (**Fig. S5**).

**Figures**

Fig. S1A.
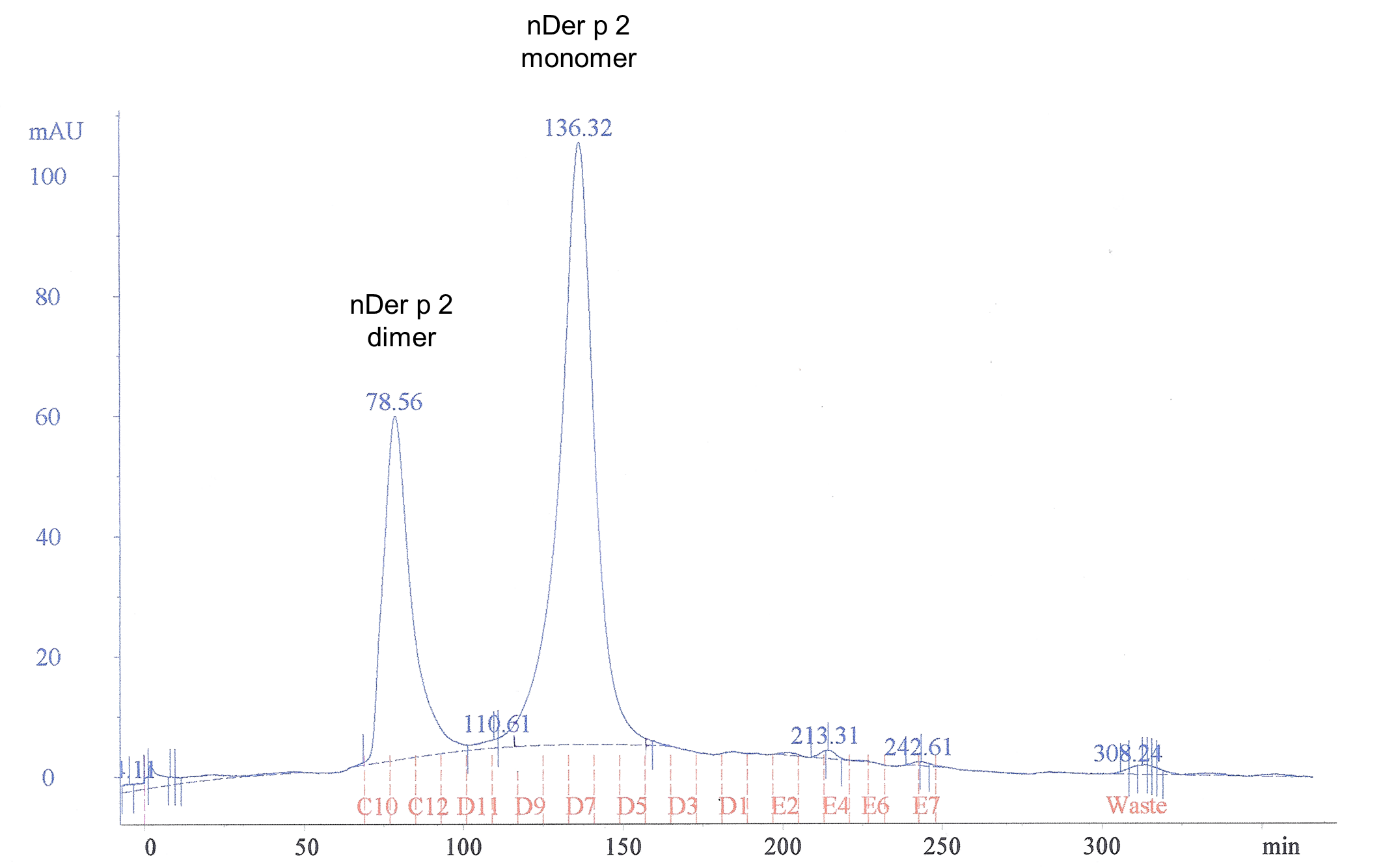


**Fig. S1B.**

**
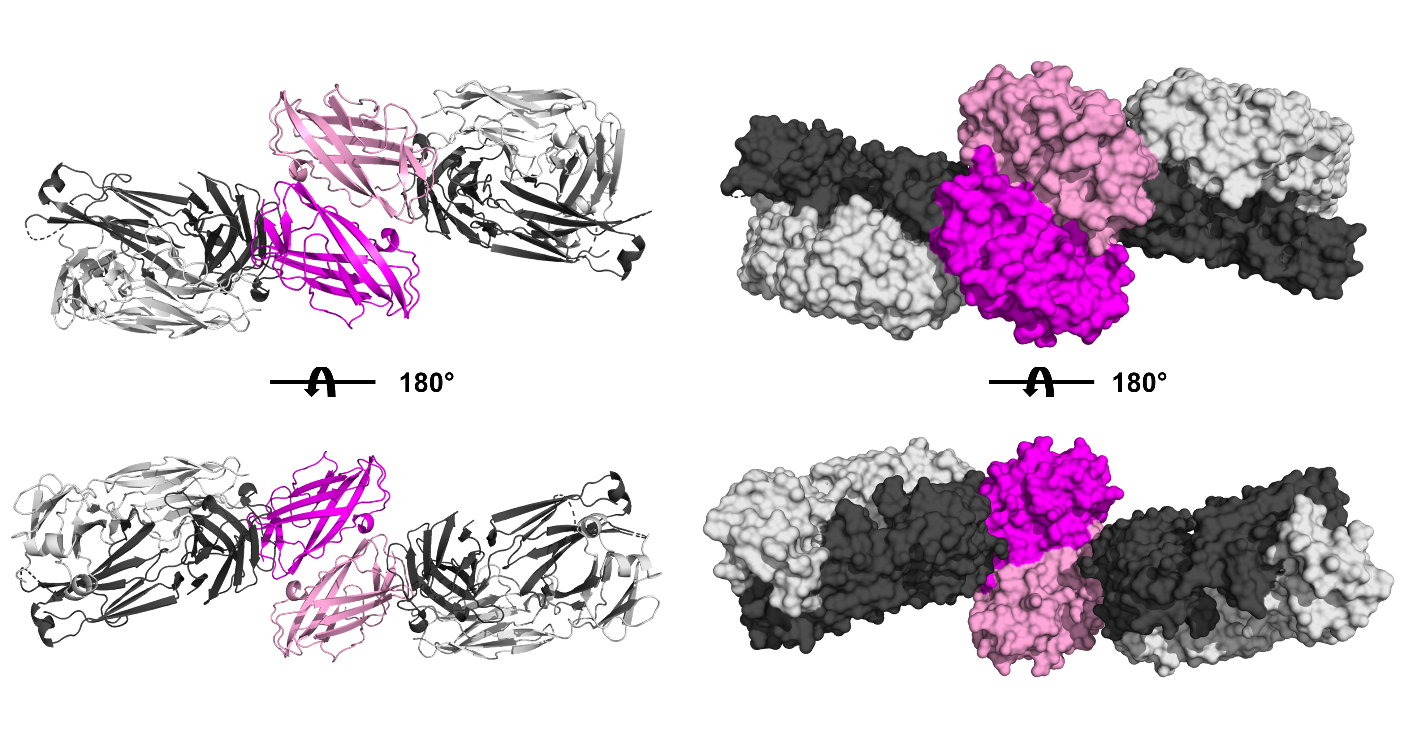
**

**Fig. S1.** Der p 2 dimerization. **A)** Elution profile of natural Der p 2 from a HiPrep 16/60 size exclusion chromatography column in 0.05 M phosphate buffer, 0.5 M NaCl, pH 7.2. Natural Der p 2 had been previously purified by affinity chromatography through a mAb 7A1 column from *D. pteronyssinus* spent dust mite culture extract before been injected (approximately 18 mg of the allergen in 2 mL) into the column. **B)** 2F10 Fab binding to a putative Der p 2.0103 dimer. The complex is shown in cartoon (left) and in surface (right) representations. Light chains are depicted in light grey, and heavy chains in dark grey. Der p 2.0103 molecules forming the dimer are shown in pick and magenta.

**Fig. S2.**


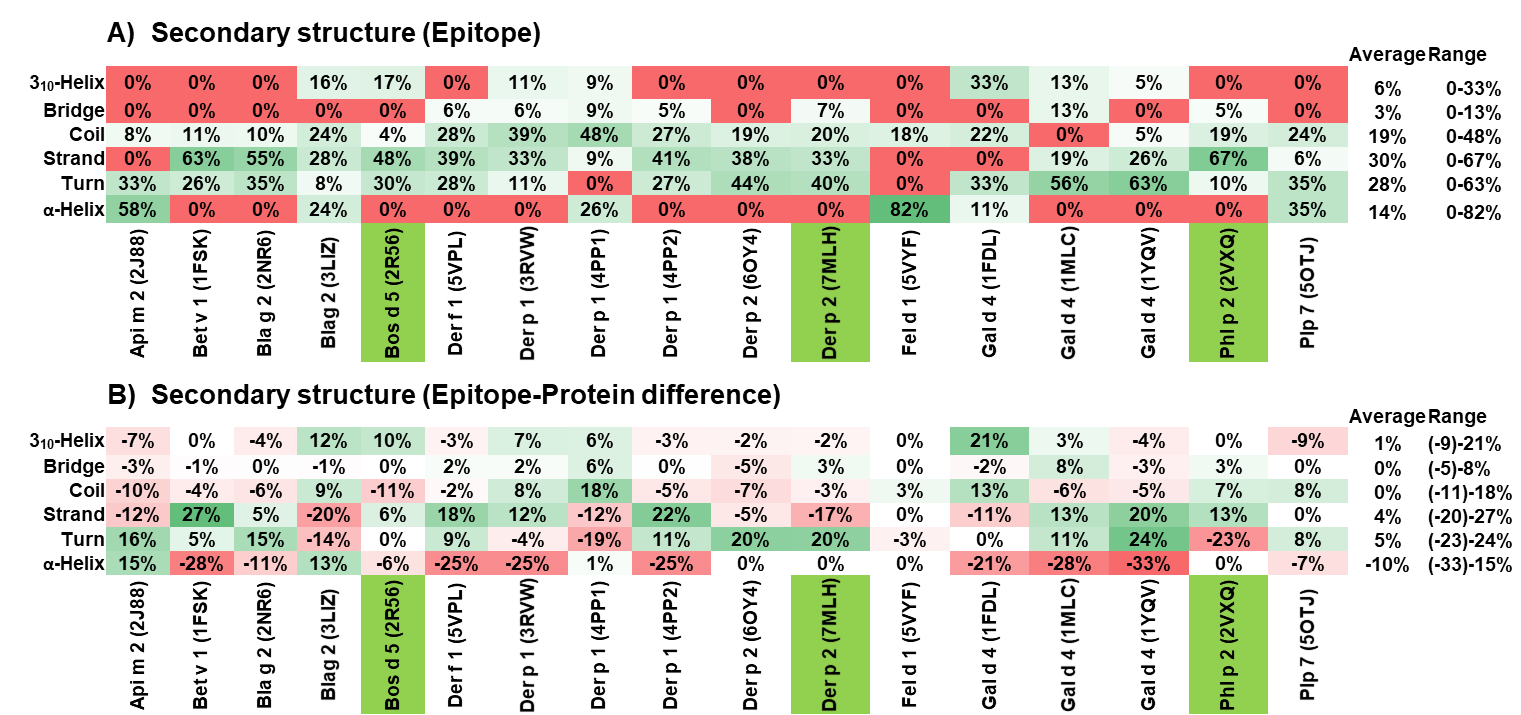


**Fig. S2.** Analysis of secondary structure elements in the epitope: **A)** in each of the 17 epitopes analyzed; Red indicates absence of a particular secondary structure element, while darker shades of green indicate a relatively large content of the secondary structure element; **B)** in each epitope in relation to the proportion of secondary structure elements found in the respective whole allergen where the epitope is. The subtraction of percentages of secondary structure elements in the epitope minus percentages in the whole protein are shown. Red indicates decrease of a particular secondary element content, white indicates no change in the secondary element content, while green indicates an increase of a particular secondary structure element in the epitope in comparison with the whole protein. Structural elements were defined by STRIDE (16). Three complexes with IgE constructs (the anti-Bos d 5 and anti-Phl p 2 from combinatorial libraries and the anti-Der p 2 with natural heavy-light chain pairing IgE) are marked in green.

Fig S3.


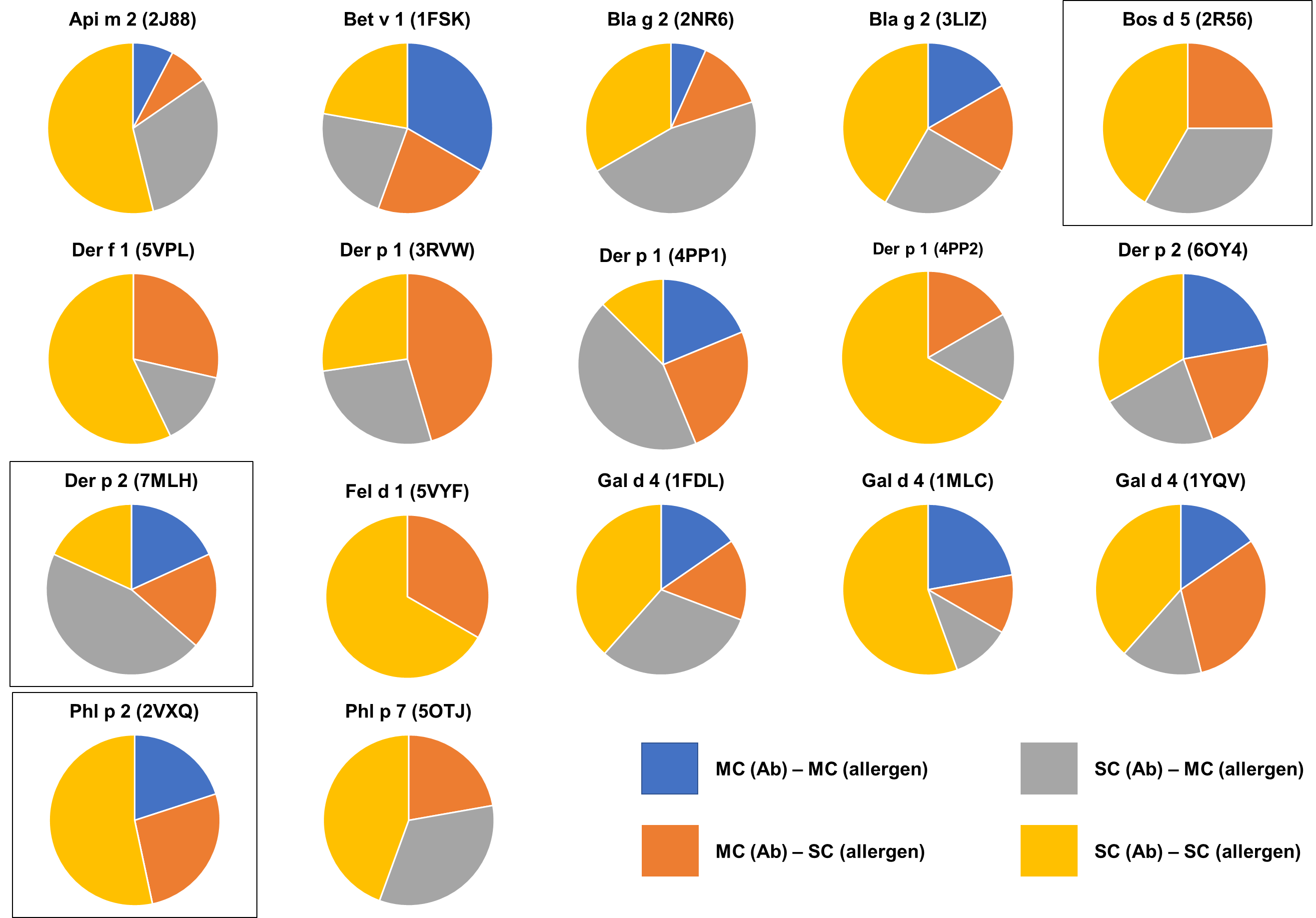


Fig. S3. Patterns of allergen-antibody hydrogen bond formation according to the involvement of main or side chains from both molecules. Seventeen allergen-antibody complexes were analyzed from which Der p 2 with IgE mAb 2F10 is the only one containing an Fab from a human IgE mAb as it occurs *in vivo.* The Bos d 2 and Phl p 2 complexes involved IgE antibody constructs obtained from phage display libraries made from PBMCs of allergic patients.

**Fig. S4A.**

**
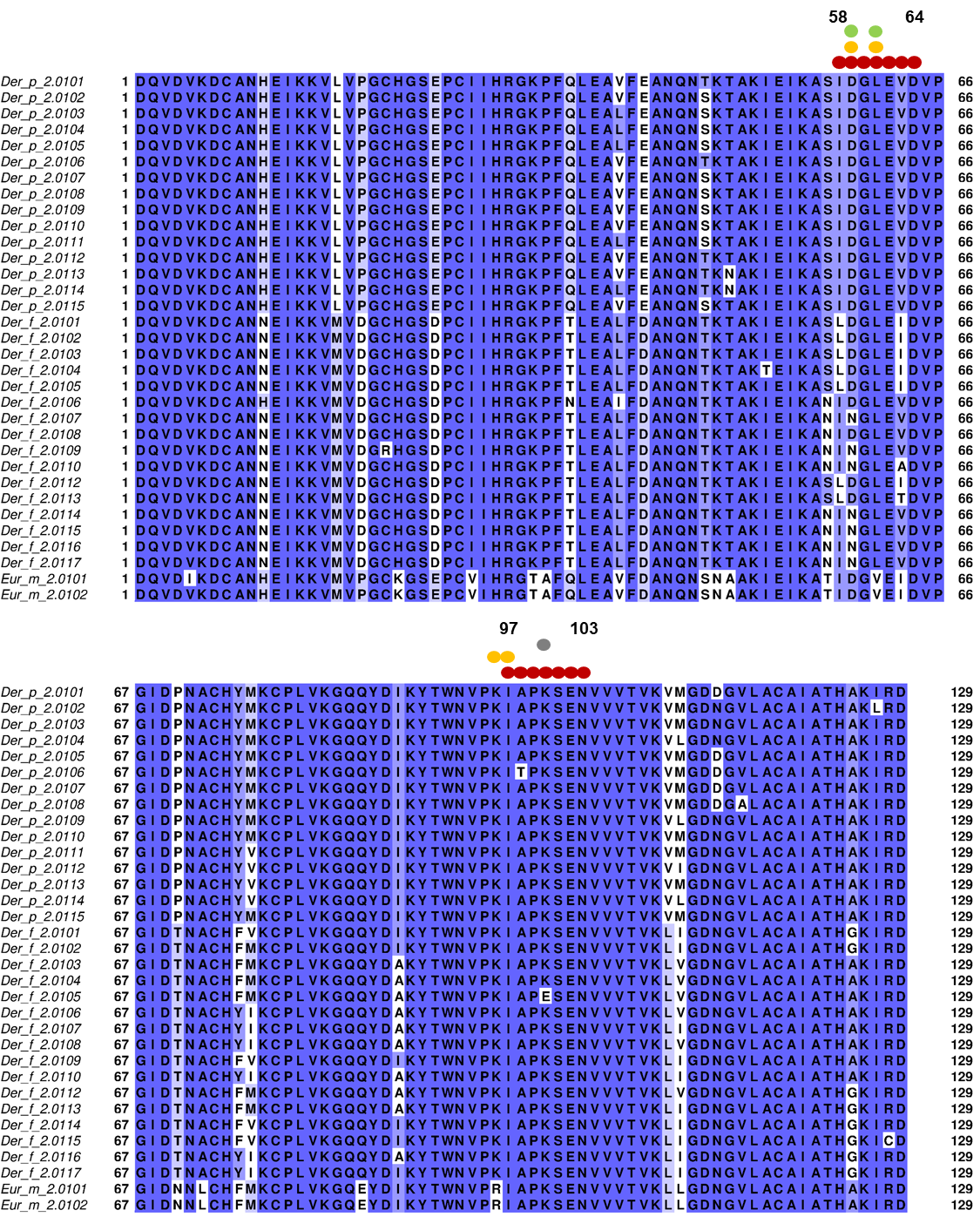
**

**Fig. S4B.**


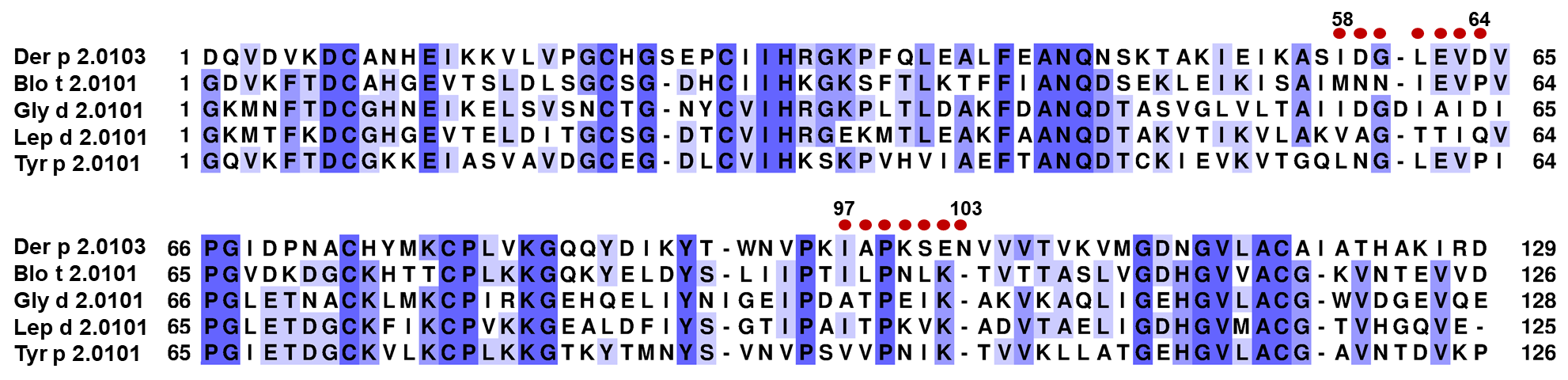


**Fig. S4C.**

**
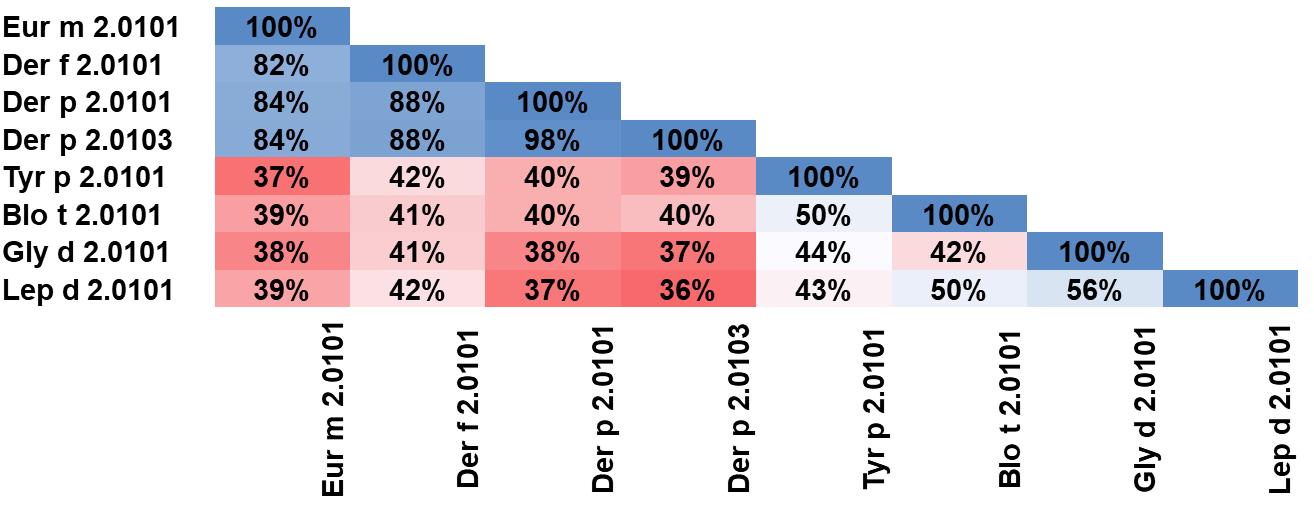
**

**Fig. S4.** Amino acid alignment of group 2 house dust mite allergens from: **A)** *Dermatophagoides pteronyssinus*, *D. farinae*, and *Euroglyphus maynei*, and **B)** *Dermatophagoides pteronyssinus* and the storage mites *Blomia tropicalis*, *Glycyphagus domesticus*, *Lepidoglyphus destructor* and *Tyrophagus putrescentiae*. Red dots indicate residues forming the 2F10 epitope*.* Green dots mark residues mutated to generate *mutant #1*. Orange dots mark residues replaced in *mutant #2*, and the grey dot marks residue that was substituted in order to generate *mutant #4.* **C)** Percent identity matrix showing amino acid sequence identity among group 2 mite allergens. Sequence identity between Der f 2, Der p 2 and Eur m 2 variants is in the 80-99% range. Sequence identities were calculated with SIAS (http://imed.med.ucm.es/Tools/sias.html).

**Fig. S5.**


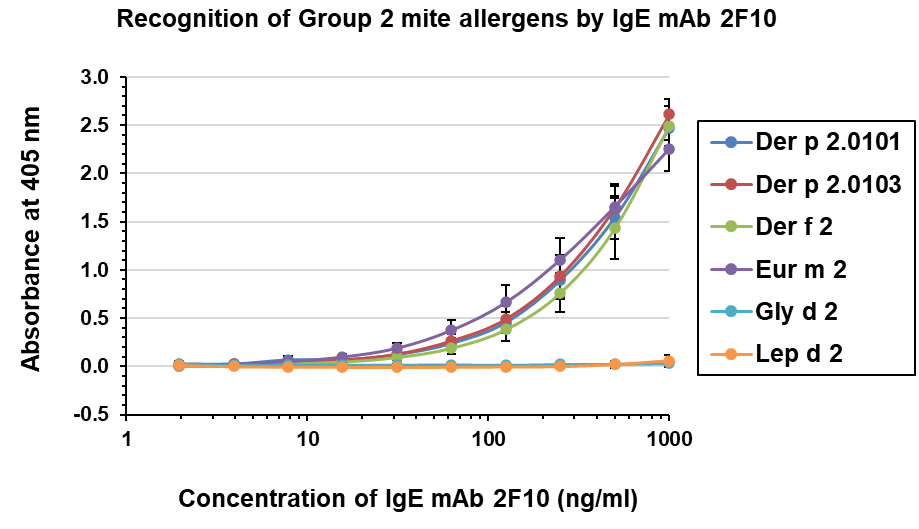


**Fig. S5.** Dose response curves of human anti-Der p 2 IgE mAb 2F10 binding to six group 2 mite allergens from different mite species. The IgE mAb 2F10 recognizes the tested group 2 allergens from house dust mites (Der p 2.0101 and Der p 2.0103 variants or isoforms, Der f 2.0103, Eur m 2.0101) but not from storage mites (Gly d 2.0101 and Lep d 2.0101). The average of 3 independent data points ± SD are represented in the graph.

Fig. S6.


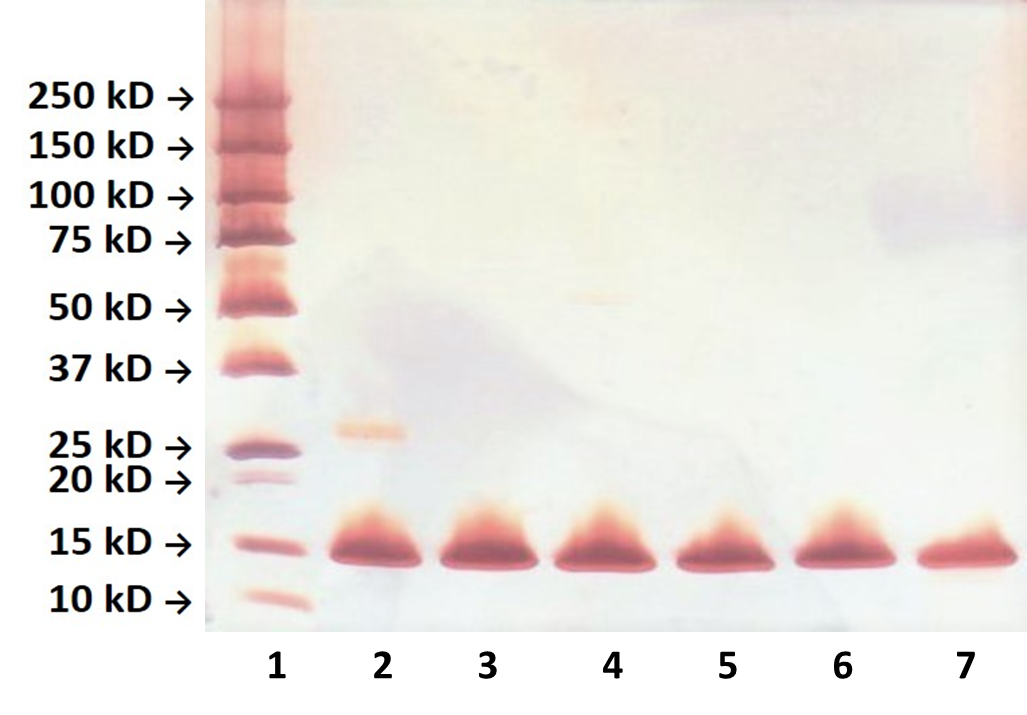


**Fig. S6.** Purified recombinant Der p 2 wildtype and 2F10 epitope mutants. SDS-PAGE silver-stained gel of the wildtype rDer p 2 (lane 2) and the 5 mutants: rDer p 2 D59A L61A (3), rDer p 2 D59A L61A + 7A1 mut (4), rDer p 2 D59K L61K (5), rDer p 2 K100D (6), and rDer p 2 D59K L61K K100D (7). Molecular weight marker is in lane 1. All allergens run at the expected MW (14 kDa) with the occasional formation of dimers in the gel at certain concentrations (as seen here for the wildtype; see also dimers in solution in Figure S1A), as previously reported for other allergens such as Der p 1 (17).

Fig. S7.


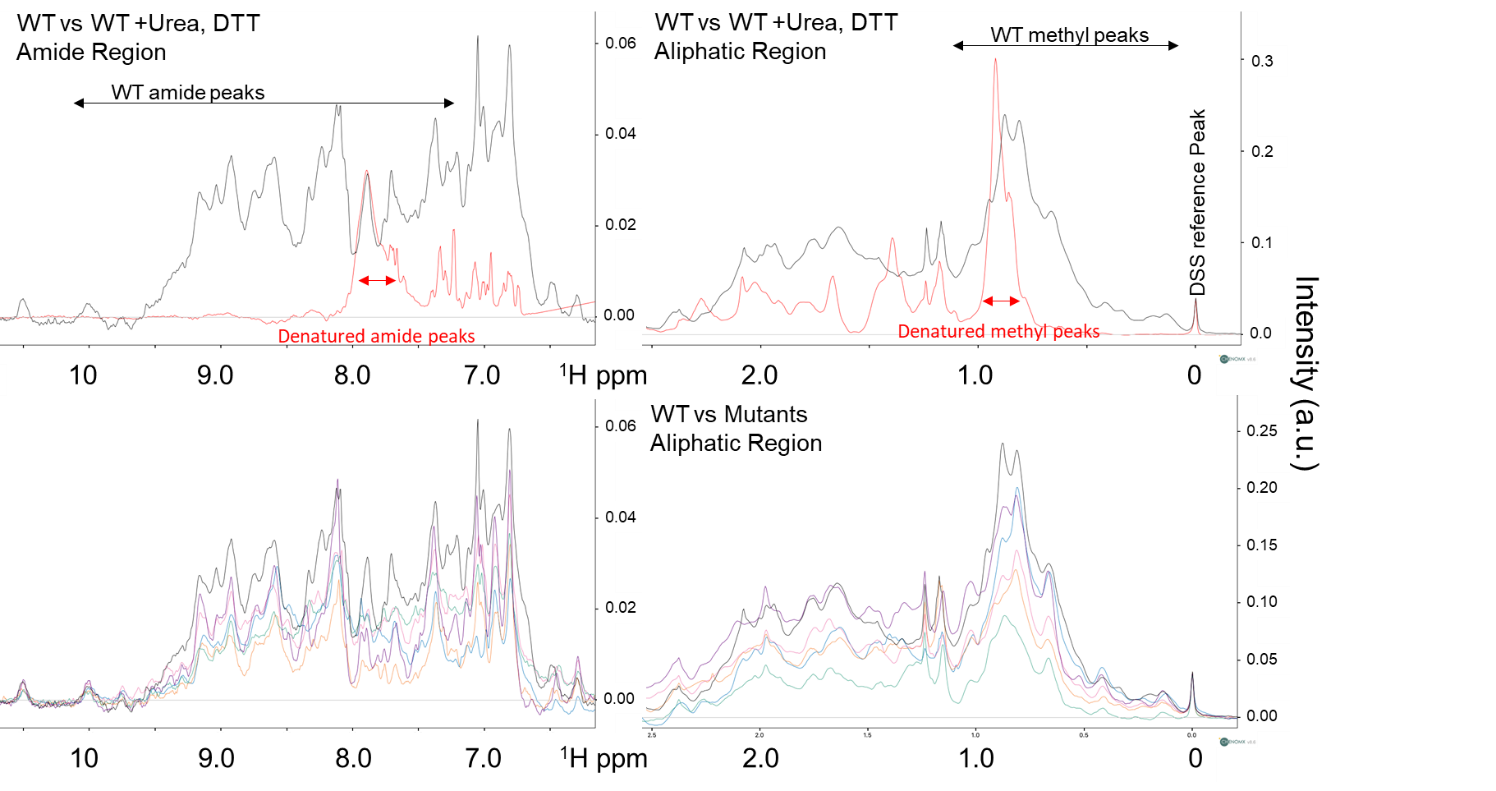


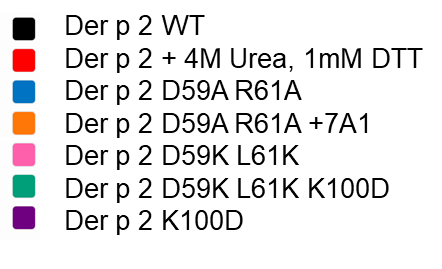


Fig. S7. NMR data demonstrates comparable folding of Der p 2 wildtype (WT) and mutant proteins. The top panels compare the WT to the urea denatured sample showing the differences between an unfolded protein (red) versus folded Der p 2 (black). The bottom panels compare the WT Der p 2 to the five mutants. The X-axis shows the chemical shift in ppm. The y-axis intensity is scaled to the reference DSS peak so that differences in the protein peak heights or total area under the curve are proportional to the protein concentration.

**Fig. S8.**


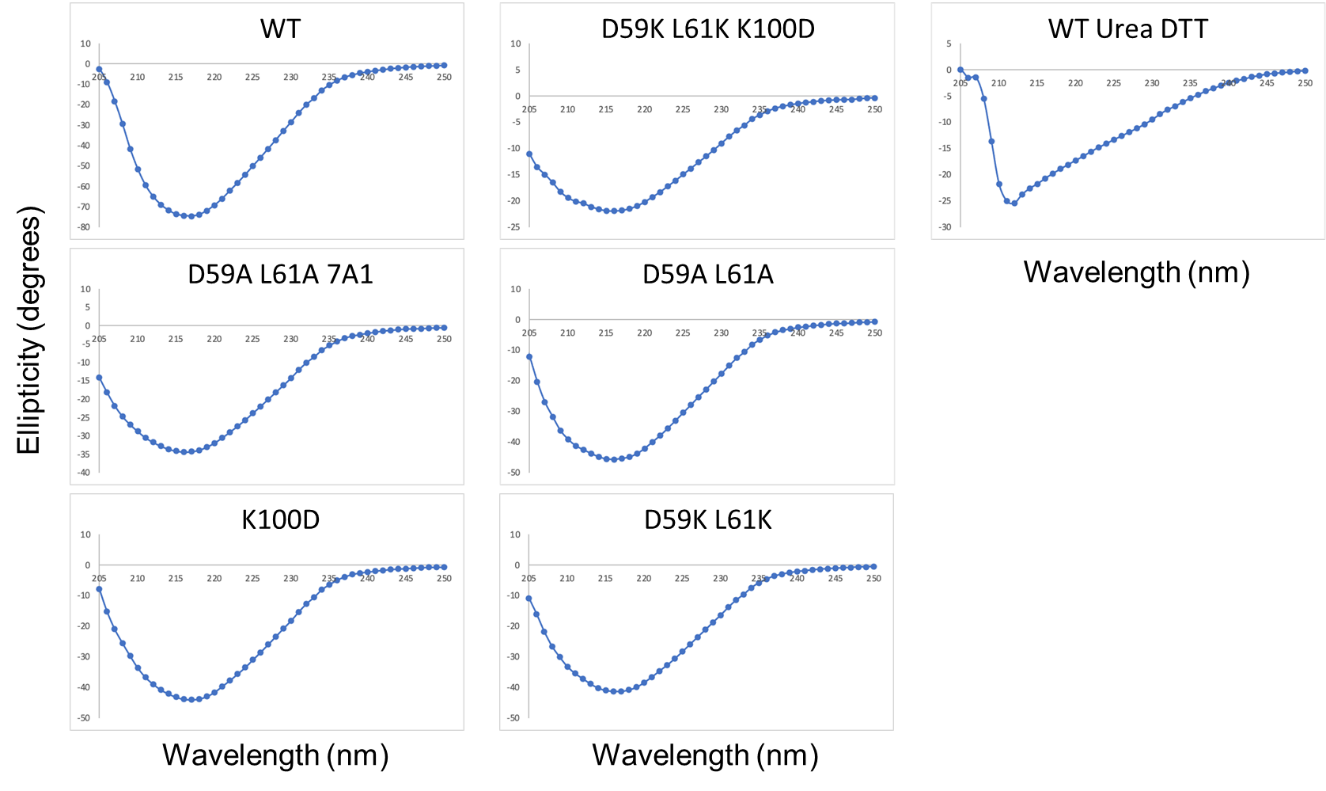


**Fig. S8.** Circular dichroism data demonstrates comparable folding of Der p 2 wildtype (WT) and mutant proteins. CD spectra for wildtype, the 5 mutants and the urea-treated Der p 2 are shown. Data was signal averaged over 4 acquisitions. The data shown resulted from subtracting the measurement acquired with the respective buffer.

**Fig. S9.**


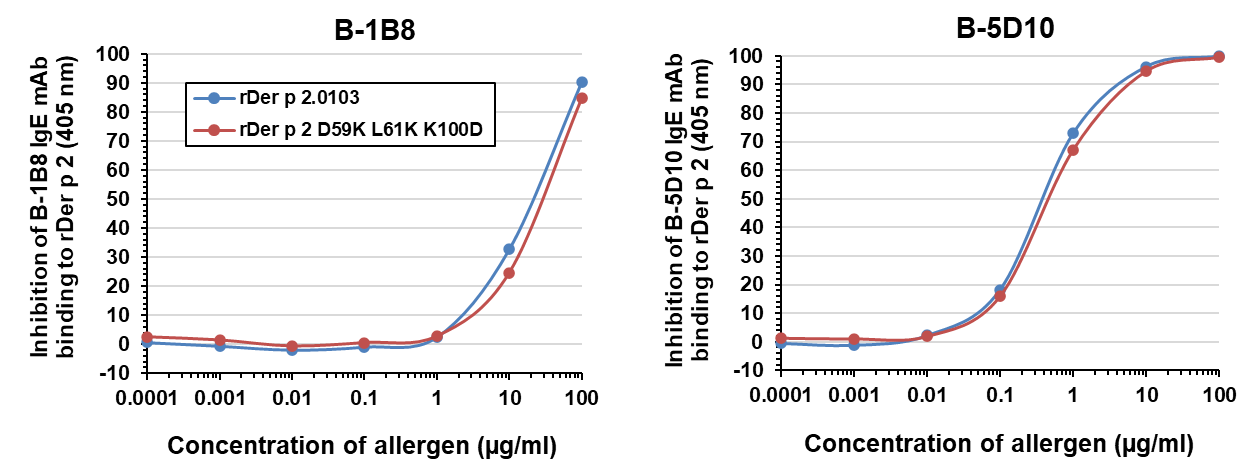


**Fig. S9.** Comparable inhibition of IgE mAb 1B8 and 5D10 to Der p 2 by Der p 2 wildtype and the 2F10 epitope triple mutant. These control experiments show that the 2F10 epitope triple mutant inhibits binding of IgE mAb 1B8 and 5D10 (that recognize different epitopes than 2F10) in an equivalent way as the wildtype Der p 2. The comparable curves for wildtype and mutant in two independent experiments indicate that the mutant folds as the wildtype, and therefore keeps the epitopes for 1B8 and 5D10 despite the mutations in the distant 2F10 epitope.

Fig. S10.


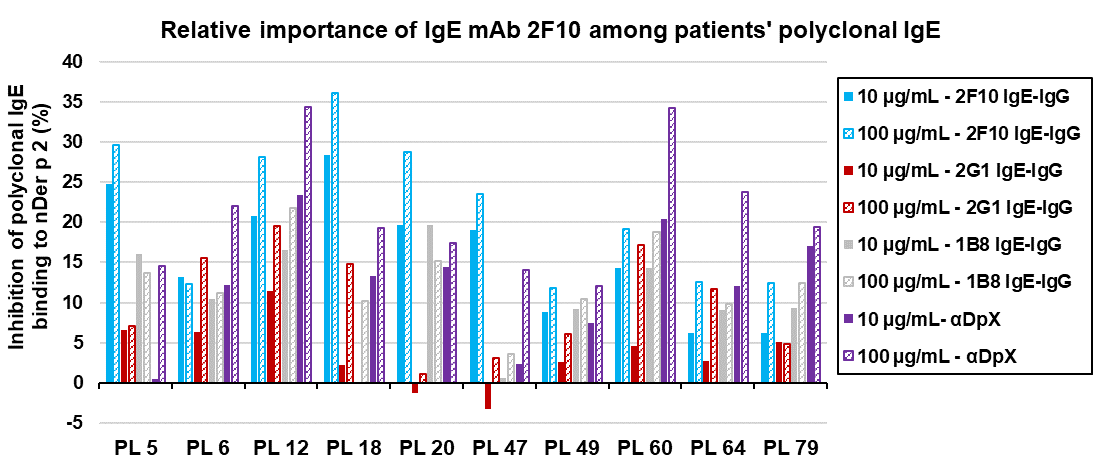


Fig. S10. Assessment of the contribution of IgE mAb 2F10 to subjects’ IgE polyclonal response. Inhibition of human polyclonal IgE antibody binding to nDer p 2 by the mAbs indicated in the figure legend was measured. Each antibody was used as inhibitor at 10 (plain bars) and 100 µg/mL (striped pattern bars). The 100 µg/mL is considered to maximally inhibit IgE antibody binding (15, 18).

**Fig. S11A.**

| **Plasma** | **Percentage of maximum inhibition** | | **Difference** | **ImmunoCAP (KU_A_/L)** |
| --- | --- | --- | --- | --- |
|  | WT | MUT* | WT-MUT** |  |
| PL 5 | 99.6 | 91.1 | 8.5 | 24.3 |
| PL 6 | 98.5 | 42.8 | 55.7 | 64.8 |
| PL 12 | 99.0 | 86.6 | 12.5 | 26.8 |
| PL 18 | 98.0 | 67.2 | 30.8 | 40.9 |
| PL 20 | 98.7 | 91.9 | 6.7 | 35.6 |
| PL 47 | 98.4 | 88.0 | 10.4 | 49.5 |
| PL 49 | 98.7 | 42.7 | 55.9 | 65.7 |
| PL 60 | 97.3 | 90.1 | 7.3 | 24.0 |
| PL 64 | 97.9 | 87.8 | 10.0 | 34.4 |
| PL 79 | 98.6 | 75.1 | 23.5 | 51.8 |
|  |  |  |  |  |
| **Average** | **98.5** | **76.3** | **22.1** | **41.8** |
| **Std dev** | **0.6** | **19.3** | **19.3** | **15.6** |
|  |  |  |  |  |
| * and ** showed high inverse correlations with the ImmunoCAP values (r = -0.86; p = 0.0016) | | | | |
|  | | | | |

**Fig. S11B.**

**
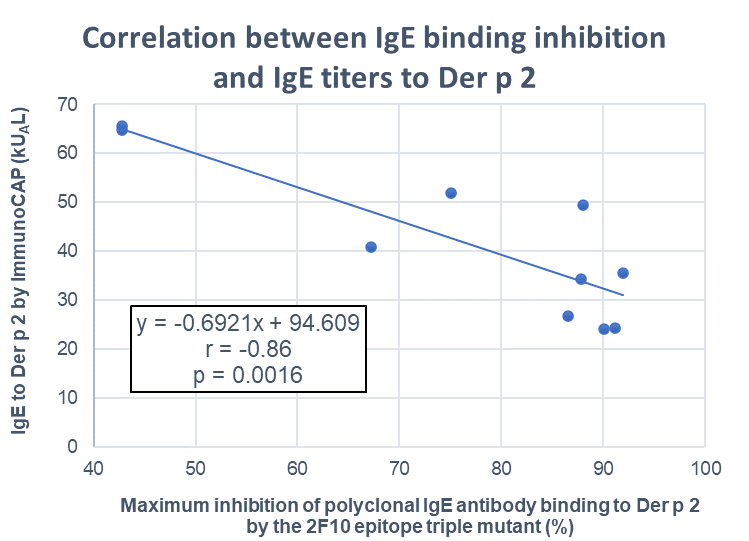
**

Fig. S11. A) Values of percentage of inhibition of polyclonal IgE antibody binding to Der p 2 by the wildtype (WT) and the 2F10 epitope triple mutant (MUT) (each at the maximum inhibitor concentration tested of 100 µg/mL), and the IgE titers measured by ImmunoCAP. B) Inverse correlation between the percentages of inhibition of polyclonal IgE antibody binding to Der p 2 by the 2F10 epitope triple mutant, and the anti-Der p 2 IgE titers measured by immunoCAP, shown in A. This was the maximum inhibition tested at 100 µg/mL of the mutant (as shown in Figure 3C).

**Table S1.** Data collection, refinement, and model validation statistics. Values for the highest resolution shells are shown in parenthesis.

| PDB accession code | 7MLH |
| --- | --- |
| **Data collection** |  |
| Diffraction source | APS, 22ID |
| Wavelength (Å) | 1.000 |
| Space group | P2_1_ |
| a, b, c (Å); β (°) | 51.8, 231.5, 51.8; 94.3 |
| Resolution range (Å) | 2.10-40.00 (2.10-2.14) |
| No. of unique reflections | 68319 (3025) |
| Completeness (%) | 96.6 (86.3) |
| Redundancy | 3.6 (3.1) |
| <I/σ(I)> | 16.9 (1.9) |
| R_meas_ | 0.113 (0.509) |
| R_p.i.m_ | 0.058 (0.269) |
| CC_1/2_ | 0.987 (0.824) |
| **Refinement** |  |
| Resolution range (Å) | 2.10-38.61 (2.10-2.15) |
| Completeness (%) | 96.4 (84.3) |
| No. of reflections, working set | 64,647 (4197) |
| No. of reflections, test set | 3560 (199) |
| Final R*_cryst_* | 0.210 (0.278) |
| Final R*_free_* | 0.247 (0.300) |
| Rmsd bonds (Å) | 0.010 |
| Rmsd angles (°) | 1.54 |
| **Ramachandran Plot** |  |
| Allowed (%) | 100 |
| Favored (%) | 98 |

| **IgE MAb** | Light  Chain | Germline Gene Segments | | | | | AA Junction | CDR3 Length | Variable Gene Mutations | | | |
| --- | --- | --- | --- | --- | --- | --- | --- | --- | --- | --- | --- | --- |
|  |  | VH | D | JH | VL | JL |  |  | VH NT | VH AA | VL NT | VL AA |
| **2F10** | κ | 1-69 | 2-2 | 4 | 1-5 | 1 | CATFRLTGYSGSGSYW | 14 | 17 | 8 | 13 | 9 |

**Table S2.** Genetic features of Der p 2-specific human IgE mAb 2F10

Antibody germline gene segment usage is shown for variable (V), diverse (D), and joining (J) regions of both the light and heavy chain based on the ImMunoGeneTics, IMGT database. The number of nucleotide and amino acid mutations are shown. The heavy and light chain sequences were found to share 94.0% and 95.7% identity, respectively, with the germline sequence. The heavy chain sequence also possessed a 6-nucleotide deletion in the third framework region, resulting in a 2 amino acid deletion.

Table S3. H-bonds formed between Der 2.0103 and mAb 2F10.

| **Der p 2.0103** | **2F10** | **CDR†** | **Distance (Å)**  **(Complex I)** | **Distance (Å)**  **(Complex II)** |
| --- | --- | --- | --- | --- |
| Asp59 (Oδ1)* | H Thr100 (Oγ1) | H-CDR3 | 2.7 | 2.7 |
| Asp59 (Oδ2)* | H Thr100 (N) | H-CDR3 | 2.7 | 2.7 |
| Asp59 (O) | L Arg95 (Nη1) | L-CDR3 | 2.7 | 2.8 |
| Asp59 (O) | L Arg95 (Nη2) | L-CDR3 | 2.9 | 2.9 |
| Gly60 (O) | L His94 (N) | L-CDR3 | 2.8 | 2.7 |
| Glu62 (N) | H Asn59 (Oδ1) | H-CDR2 | 2.8 | 2.8 |
| Asp64 (N) | H Phe55 (O) | H-CDR2 | 3.0 | 2.9 |
| Lys100 (N) | H Tyr102 (Oη) | H-CDR3 | 3.2 | 3.4 |
| Lys100 (O) | H Gly101 (N) | H-CDR3 | 3.2 | 3.1 |
| Glu102 (N) | H Thr100 (Oγ1) | H-CDR3 | 2.9 | 2.9 |
| Asn103 (Nδ2)* | L His92 (O) | L-CDR2 | 3.0 | 3.0 |

*Side-chain of this epitope residue interacts with antibody

^†^CDR were estimated using the software PyIgClassify (19).

**Table S4.** IgE mAb 2F10 epitope mutants.

*Mutant #1*: Asp59 and Leu61 to alanines (rDer p 2 D59A L61A)

*Mutant #2*: Asp59 and Leu61 to alanines

plus 7A1 epitope mutations K96E-I97E (D59A L61A + 7A1 mut)

*Mutant #3*: Asp59 and Leu61 to lysines (rDer p 2 D59K L61K)

*Mutant #4*: Lys100 to aspartate (rDer p 2 K100D)

*Mutant #5*: Combination of *mutants #3* and *4* (rDer p 2 D59K L61K K100D)

**SI References**

1. N. L. Bernasconi, E. Traggiai, A. Lanzavecchia, Maintenance of serological memory by polyclonal activation of human memory B cells. *Science* **298**, 2199-2202 (2002).

2. E. Traggiai *et al.*, An efficient method to make human monoclonal antibodies from memory B cells: potent neutralization of SARS coronavirus. *Nat Med* **10**, 871-875 (2004).

3. M. A. Wurth *et al.*, Human IgE mAbs define variability in commercial Aspergillus extract allergen composition. *JCI Insight* **3** (2018).

4. G. A. Mueller *et al.*, Mapping human monoclonal IgE epitopes on the major dust mite allergen Der p 2. *J Immunol* **205**, 1999-2007 (2020).

5. Z. Otwinowski, W. Minor, Processing of X-ray diffraction data collected in oscillation mode. *Method. Enzymol* **276**, 307-326 (1997).

6. A. Vagin, A. Teplyakov, Molecular replacement with MOLREP. *Acta Crystallogr. D. Biol Crystallogr* **66**, 22-25 (2010).

7. W. Minor, M. Cymborowski, Z. Otwinowski, M. Chruszcz, HKL-3000: the integration of data reduction and structure solution-from diffraction images to an initial model in minutes. *Acta Crystallogr. D. Biol Crystallogr* **62**, 859-866 (2006).

8. M. D. Winn *et al.*, Overview of the CCP4 suite and current developments. *Acta Crystallogr. D. Biol Crystallogr* **67**, 235-242 (2011).

9. P. Emsley, K. Cowtan, Coot: model-building tools for molecular graphics. *Acta Crystallogr. D. Biol Crystallogr* **60**, 2126-2132 (2004).

10. G. N. Murshudov *et al.*, REFMAC5 for the refinement of macromolecular crystal structures. *Acta Crystallogr. D. Biol Crystallogr* **67**, 355-367 (2011).

11. I. W. Davis *et al.*, MolProbity: all-atom contacts and structure validation for proteins and nucleic acids. *Nucleic Acids Res* **35**, W375-383 (2007).

12. Anonymous, The PyMOL molecular graphics system, Version 2.0 Schrödinger, LLC.

13. E. Krissinel, K. Henrick, Inference of macromolecular assemblies from crystalline state. *J Mol Biol* **372**, 774-797 (2007).

14. M. C. Lawrence, P. M. Colman, Shape complementarity at protein/protein interfaces. *J Mol. Biol* **234**, 946-950 (1993).

15. J. Glesner *et al.*, A human IgE antibody binding site on Der p 2 for the design of a recombinant allergen for immunotherapy. *J Immunol* **203**, 2545-2556 (2019).

16. D. Frishman, P. Argos, Knowledge-based protein secondary structure assignment. *Proteins* **23**, 566-579 (1995).

17. J. Glesner *et al.*, Antigenic determinants of Der p 1: specificity and cross-reactivity associated with IgE antibody recognition. *J. Immunol* **198**, 1334-1344 (2017).

18. M. Li *et al.*, Carbohydrates contribute to the interactions between cockroach allergen Bla g 2 and a monoclonal antibody. *J Immunol* **186**, 333-340 (2011).

19. J. Adolf-Bryfogle, Q. Xu, B. North, A. Lehmann, R. L. Dunbrack, Jr., PyIgClassify: a database of antibody CDR structural classifications. *Nucleic Acids Res* **43**, D432-438 (2015).
